# Effects of repeated infections with non-typeable *Haemophilus influenzae* on lung in vitamin D deficient and smoking mice

**DOI:** 10.1186/s12931-022-01962-6

**Published:** 2022-03-02

**Authors:** Jef Serré, Ajime Tom Tanjeko, Carolien Mathyssen, Tobias Heigl, Annelore Sacreas, Dana Paulina Cook, Erik Verbeken, Karen Maes, Jan Verhaegen, Charles Pilette, Jeroen Vanoirbeek, Conny Gysemans, Chantal Mathieu, Bart Vanaudenaerde, Wim Janssens, Ghislaine Gayan-Ramirez

**Affiliations:** 1 Laboratory of Respiratory Diseases and Thoracic Surgery (BREATHE), Department of Chronic Diseases and Metabolism (CHROMETA), KU Leuven, Herestraat 49, O&NI bis, box 706, 3000 Leuven, Belgium; 2 Clinical and Experimental Endocrinology (CEE), Department of Chronic Diseases and Metabolism (CHROMETA), KU Leuven, Leuven, Belgium; 3 Translational Cell & Tissue Research, Department of Imaging & Pathology, KU Leuven, Leuven, Belgium; 4 Laboratory of Clinical Bacteriology and Mycology, Department of Microbiology, Immunology and Transplantation, KU Leuven, Leuven, Belgium; 5 Institute of Experimental & Clinical Research, Pole of Pneumology, ENT and Dermatology, and Cliniques Universitaires Saint-Luc, Department of Pulmonology, Université Catholique de Louvain (UCL), Brussels, Belgium; 6 Centre of Environment and Health, Department of Public Health and Primary Care, KU Leuven, Leuven, Belgium

**Keywords:** NTHi, Vitamin D deficiency, Immunoglobulin, Cigarette smoke-exposure, COPD

## Abstract

**Background:**

In chronic obstructive pulmonary disease (COPD), exacerbations cause acute inflammatory flare-ups and increase the risk for hospitalization and mortality. Exacerbations are common in all disease stages and are often caused by bacterial infections e.g., non-typeable *Heamophilus influenzae* (NTHi). Accumulating evidence also associates vitamin D deficiency with the severity of COPD and exacerbation frequency. However, it is still unclear whether vitamin D deficiency when combined with cigarette smoking would worsen and prolong exacerbations caused by repeated infections with the same bacterial strain.

**Methods:**

Vitamin D sufficient (VDS) and deficient (VDD) mice were exposed to nose-only cigarette smoke (CS) for 14 weeks and oropharyngeally instilled with NTHi at week 6, 10 and 14. Three days after the last instillation, mice were assessed for lung function, tissue remodeling, inflammation and immunity. The impact of VDD and CS on inflammatory cells and immunoglobulin (Ig) production was also assessed in non-infected animals while serum Ig production against NTHi and dsDNA was measured in COPD patients before and 1 year after supplementation with Vitamin D3.

**Results:**

VDD enhanced NTHi eradication, independently of CS and complete eradication was reflected by decreased anti-NTHi Ig’s within the lung. In addition, VDD led to an increase in total lung capacity (TLC), lung compliance (Cchord), MMP12/TIMP1 ratio with a rise in serum Ig titers and anti-dsDNA Ig’s. Interestingly, in non-infected animals, VDD exacerbated the CS-induced anti-NTHi Ig’s, anti-dsDNA Ig’s and inflammatory cells within the lung. In COPD patients, serum Ig production was not affected by vitamin D status but anti-NTHi IgG increased after vitamin D3 supplementation in patients who were Vitamin D insufficient before treatment.

**Conclusion:**

During repeated infections, VDD facilitated NTHi eradication and resolution of local lung inflammation through production of anti-NTHi Ig, independently of CS whilst it also promoted autoantibodies. In COPD patients, vitamin D supplementation could be protective against NTHi infections in vitamin D insufficient patients. Future research is needed to decipher the determinants of dual effects of VDD on adaptive immunity.

***Trail registration*:**

ClinicalTrials, NCT00666367. Registered 23 April 2008, https://www.clinicaltrials.gov/ct2/show/study/NCT00666367.

**Supplementary Information:**

The online version contains supplementary material available at 10.1186/s12931-022-01962-6.

## Background

Chronic obstructive pulmonary disease (COPD) is characterized by an irreversible airflow limitation associated with persistent respiratory symptoms. Currently, COPD is the third leading cause of mortality worldwide [1]. One of its major risk factors is cigarette smoking, which induces an abnormal chronic inflammatory response in the lungs that drives the pathophysiology of COPD.

COPD is complicated by acute exacerbations, acute flare-ups of the disease that worsen respiratory symptoms and require a change in medication or even hospitalization with a high risk for mortality [2]. Exacerbations are mostly caused by bacterial infections such as non-typeable *Haemophilus influenza* (NTHi) (20–30%), *Streptococcus pneumonia* (10–15%), *Moraxella catarrhalis* (10–15%) and *Pseudomonas aeruginosa* (10–15%) that colonize the lower airways predisposing COPD patients to repeated infections, not necessarily by the same strain [3]. In addition, cigarette smoking will create an optimal environment for lower airway colonization and infections through several mechanisms: 1) by altering the airway epithelium and reducing mucociliary clearance; 2) by reducing phagocytosis of micro-organisms [4] and 3) by impairing efferocytosis of apoptotic cells [5]. Exacerbations occur at all stages of the disease however, the frequency and severity increases as the disease progresses (e.g. for GOLD 2 and 4, respectively: 0.85 and 2 exacerbations/year with 7% and 33% of the exacerbations requiring hospitalization) [6]. Frequent exacerbators (≥ 2 exacerbations per year) are also found in all disease severity classes [6]. Exacerbations are associated with a worsening of the disease with a deterioration of lung function and symptoms, a severe loss in functional status and impaired quality of life, and are a major cause of emergency hospitalization with an enhanced risk of mortality [7]. It is therefore warranted to find strategies to limit exacerbations in COPD patients.

In recent years, accumulating evidence suggests a link between vitamin D deficiency [25-hydroxyvitamin D (25-OHD) serum levels < 20 ng/ml (< 50 nmol/l)] and COPD severity [8–10]. Apart from its classical role in calcium homeostasis, vitamin D is also implicated in regulating inflammation [11, 12], antimicrobial peptide production [4], T-cell development [11, 13], antigen presenting cell maturation [14, 15], as well as lung development [16, 17] and remodeling [18, 19]. In Europe, vitamin D deficiency is highly prevalent and affects approximately 48–89% of the elderly (> 65 years) [20, 21] but is more frequent in COPD patients where it increases with disease severity [8]. In addition, clinical studies indicated an inverse relationship between 25-OHD serum levels and upper and lower respiratory tract infections [22, 23], community-acquired pneumonia [24] and tuberculosis [25]. Observational studies revealed that severe vitamin D deficiency (25-OHD serum levels < 10 ng/ml) correlated with frequent exacerbations in COPD patients [26, 27]. Interestingly, a 1 year oral supplementation with vitamin D led to a 40% decrease in exacerbation incidence but only in a small group of COPD patients with severe vitamin D deficiency [10]. This was further supported by a recent meta-analysis, indicating that vitamin D supplementation only reduced the rate of exacerbations in severe vitamin D deficient patients, but not in patients with baseline serum 25-OHD levels > 10 ng/ml [9].

Few animal models have been developed to investigate the relationship between vitamin D deficiency and respiratory infections. Hampering vitamin D functionality by deletion of the vitamin D receptor (VDR) in mice impaired the pulmonary epithelial barrier integrity by disrupting the tight and adherens junctions [17] and, when combined with lipopolysaccharide (LPS) infection, it resulted in an increased alveolar permeability and inflammation compared to wild-type mice [28]. On the other hand, vitamin D deficient mice have displayed a defective resistance against. *Aspergillus fumigates*, with increased mortality and inflammatory response [29]. However, in a similar set-up no dysfunction of the host defense was observed following an infection with *Pseudomonas aeruginosa* or *Streptococcus pneumoniae* [30], suggesting that the effects might be inoculation- and strain-specific. Interestingly, while cigarette smoke (CS) combined with heat killed NTHi exposure led to an excessive inflammatory response and airway COPD characteristics after 3 months of CS exposure in mice [31], we surprisingly found that an acute airway infection with NTHi resulted in a faster eradication in vitamin D deficient mice irrespective of 6 weeks CS exposure [32]. Vitamin D deficiency was also associated with an increased production of matrix metalloproteinase-12 (MMP-12), indicative of increased proteolysis in the lungs [32]. It is however still unclear whether vitamin D deficiency would worsen the chronic inflammation induced by CS exposure and prolong the exacerbations when exposed to repeated viable NTHi infections, as in frequent COPD exacerbators. This hypothesis has been addressed in a smoking mouse model designed to mimic cigarette smoke-induced chronic inflammation, in which mice were made vitamin D deficient through a diet and subjected to three repeated infections with NTHi. Lung function as well as inflammation and immunity were examined in this model.

## Materials and methods

### Study design

This study consists out of three series of experiments: two studies in mice and some measurements performed in patients with COPD. In the mice study, the impact of vitamin D deficiency combined or not with cigarette smoking on inflammatory cells and immunoglobulin production was first addressed on non-infected animals (series 1). Then, the effects of vitamin D deficiency and smoking were examined in animals repeatedly infected with NTHi in whom lung function and histopathology, bacterial clearance, inflammatory mediators, lymph node activation and immunoglobulin (Ig) production were measured (series 2). Finally, Ig production against NTHi and DNA was measured in serum of COPD patients with different baseline 25-OHD serum levels.

### Animal model

#### Animals

Three week-old male C57Bl/6JolaH mice were fed with a vitamin D deficient (VDD; n = 32 and n = 30 mice for series 1 and series 2, respectively) (TD.87095 containing 20% w/w lactose, 2% w/w calcium and 1.25% w/w phosphorus, Envigo Teklad custom diet, Madison, Wis. USA) or vitamin D sufficient diet (VDS; n = 32 and n = 26 mice for series 1 and series 2, respectively) (Ssniff Bioservices, Uden, The Netherlands) and were kept in an ultraviolet light-free environment during the whole study, as described in [32]. Mice were housed in individually ventilated cages with a 12/12 h light–dark cycle.

#### Exposure to cigarette smoking or room air

At the age of eight weeks, mice were randomly divided into two separate groups: nose-only CS- and air-exposed groups using a nose-only exposure system (InExpose system, Scireq). Mice were placed in soft restraints connected to an exposure tower and a computer-controlled puff was generated every minute to expose mice to 10 s CS followed by 50 s of room air while control mice were exposed to room air. CS-exposed mice were first acclimatized for one week with an increasing number of cigarettes with filter (3R4F research cigarettes with filter, Kentucky Tobacco R&D center, university of Kentucky) per session, until the limit of 6 cigarettes per session was reached while control mice were exposed to ambient air and kept in soft restraints for the same duration as established previously [32]. Afterwards, mice were exposed twice a day to CS or to ambient air, 5 days/week for 14 weeks (16 controls and 16 smokers for series 1; 13/15 controls and 13/15 smokers for series 2). With this protocol, particle density per session averaged 159 ± 19 mg/m^3^ (Microdust, Casella CEL, Bedford, UK) in the CS group.

#### Induction of repeated infections in series 2 only

**Fig. 1:**
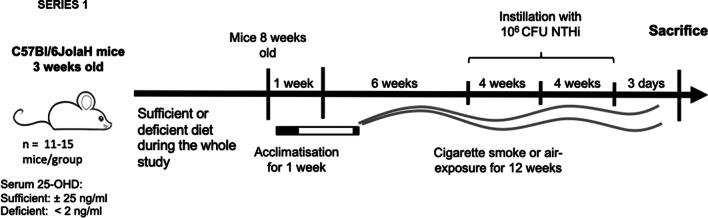
Experimental mouse model with three repeated NTHi infections (series 2): 3-week-old male mice were fed during 5 weeks with a vitamin D deficient or sufficient diet and were maintained under respective diets during the whole study period. At the age of 8 weeks, mice were acclimatized for 1 week to cigarette smoke or ambient air in soft restrains and then exposed to cigarette smoke (6 cigarettes/day 5 day/week) or room air during the study period. In series 2, at week 6 of exposure, mice were infected oropharyngeally with 10^6^ CFU/ml NTHi and maintained one week in quarantine with no exposure and then CS or air exposure were resumed. They were infected again on week 10 and 14 of exposure and measurements were performed 72 h after the last infection

On week 6 of CS or air-exposure, mice were oropharyngeally infected with 1 × 10^6^ colony forming units (CFU) NTHi in a total volume of 50 μl PBS (kindly provided by J.V.) and subsequently repeated on week 10 and 14 (Fig. 1). Before infection, mice were anaesthetized intraperitoneally (i.p.) with a mixture of xylazine (4.25 mg/kg, Rompun®, Bayer, Belgium) and ketamine (65 mg/kg, Ketalar® Pfizer, Belgium). After each infection mice were kept in quarantine for 1 week during which they were not exposed to CS. CS or air exposure sessions were resumed after this 1-week of quarantine.

#### Measurements

For series 1, inflammatory cells in broncho-alveolar lavage (BAL) and immunoglobulin production in BAL and serum were measured while for series 2, lung function and histopathology, bacterial clearance, inflammatory mediators, lymph node activation and Ig production were assessed 72 h after the last infection. Levels of serum 25-OHD were determined in the two mouse studies.

### Patients characteristics

Serum samples from 137 vitamin D-naïve COPD participants were included in our study. These patients were issued from a previous single-center, double-blind, randomized, placebo-controlled trial (RCT) (University hospitals Leuven, Belgium), approved by the local ethics committee of University Hospital of Leuven (S50722, EudraCT number: 2007-004755-11) and ClinicalTrials.gov (NCT00666367). In this RCT study, the patients were treated with either a high-dose of vitamin D3 (100,000 IU, D-Cure) or a placebo (arachidic oil) every 4 weeks for one year. Details of the vitamin D intervention trial have been previously published [10]. In this *post-hoc* analysis, we assessed total IgG and IgG against NTHi and DNA in serum of placebo or vitamin D-treated COPD patients. Patients were matched according to gender, current smokers, GOLD stage, serum 25-OHD level, Forced Expiratory Volume in 1 s (FEV_1_), Forced Vital Capacity (FVC) and FEV_1_/FVC (Additional file 10: Table S2). Participants from the RCT did not receive any antibiotics or oral steroid treatment for an acute exacerbation during the 1-year study period. A subgroup analysis was also performed by dividing the participants into severe vitamin D deficient (< 10 ng/ml 25-OHD), vitamin D deficient (< 20 ng/ml 25-OHD) and vitamin D insufficient (< 30 ng/ml) patients. Vitamin D sufficient patients (> 30 ng/ml 25-OHD) were excluded, because of insufficient numbers for reliable statistics (n = 7, Additional file 10: Table S2).

### Animal model measurements:

#### Lung function (series 2)

Mice were anaesthetized i.p. with a Xylazine (10 mg/kg, Rompun®, Bayer, Belgium), Ketamine (100 mg/kg, Ketalar®, Pfizer, Belgium) and Acepromazine (3 mg/kg, Placivet®, Kela, Belgium) mixture 3 days after the last NTHi infection. Sedated mice were tracheotomized and placed in the whole-body plethysmograph (Force Pulmonary Maneuvers, Buxco®) to measure total lung capacity (TLC) and chest wall compliance (Cchord) [33].

#### Broncho-alveolar lavage (both series)

BAL was performed with 500 μl and 3 times 1000 μl of saline (B. Braun Medical, Diegem, Belgium). The supernatant of the first fraction was collected for cytokine analysis by centrifugation (10 min, 1000 g, 4 °C) (series 2 only), while total cell count was measured on all pooled cell fractions using a Bürker hemocytometer with trypan blue. Differential cell counts were determined after centrifugation (6 min, 300 g) of the BAL cells onto microslides with cytospins (Shandon, TechGen, Zellik, Belgium) and staining with Diff-Quick® (Medical Diagnostics, Düdingen, Germany). On each slide, 100 cells were counted 3 times to determine the various differentiated cells.

For series 2, bacterial clearance was measured in BAL only as we previously showed that the time course of bacterial load changes post NTHi infection followed a similar pattern between BAL and lung homogenate. Importantly, bacteria were still detectable in BAL after 64 h while this was not the case in lung homogenate [32]. To address bacterial clearance, viable NTHi was assessed at sacrifice, by plating serial dilutions onto *Haemophilus* agar plates. The number of colonies were counted 24 and 48 h after incubation (37 °C) and expressed as the number of CFU per milliliter of BAL.

#### 25-OHD serum levels (both series)

Blood collected from the vena cava was kept at room temperature for 1 h and centrifuged at 1500 g for 15 min (4 °C). Serum was collected and stored at − 80 °C. Serum 25-OHD levels were measured by liquid chromatography tandem-mass spectrometry (LC–MS/MS) (LC, Shimadzu, and MS, Qtrap 5500, Sciex) at the laboratory of medicine, UZ Leuven. Samples were extracted with methanol mixture spiked with internal standard 25-OH Vit-D3-d6.

#### Histopathology of the lungs (series 2)

The heart-left lung block was fixed for 24 h under a constant hydrostatic pressure (20–25 cmH_2_O) with 6% paraformaldehyde. The left lung was isolated and cut in three transversal pieces (apical, center and basal), dehydrated and embedded in paraffin. Transversal lung slides were stained with H&E and scored for inflammation in a blinded manner by a lung pathologist (E.V.). Each compartment of the lung was scored separately e.g. broncho-vascular bundle, parenchymal and venous compartment for lymphoid aggregates and diffuse inflammation. Scoring ranged from 0 to 3: 0 = normal, 0.5 = minimal, 1 = mild, 2 = moderate and 3 = severe. Airspace enlargement was quantified by measuring the mean linear intercept (Lm) in 25 fields over the whole lung using an in-house macro in ImageJ.

#### Expression of proteases/antiproteases (series 2)

The right lung was removed, snap frozen in liquid nitrogen and stored at − 80 °C for RNA extraction. After homogenization in TRIzol® (ThermoFisher scientific, Life Technologies, Waltham, MA USA), total RNA was extracted with chloroform and purified with RNAeasy mini kit (Qiagen, Leudsen, Netherlands) according to manufacturer’s instructions.  One µg of RNA was reverse transcribed with random hexametric primers and Superscript III reverse transcriptase was used for cDNA synthesis at 42 °C for 50 min, followed by 15 min incubation at 70 °C. The quantitative Real-Time PCR amplifications (qRT-PCR) were performed with 10 ng cDNA and Platinum®SYBR® Green qPCR SuperMix-UDG (ThermoFisher scientific) using a thermal cycler (Eco Real-Time PCR system, Illumina). Threshold data was normalized using a housekeeping gene, Ribosomal Protein L27 (RPL27), and groups were compared using the comparative cycle threshold (∆∆CT) method. Primers are given in Additional file 10: Table S1.

#### Inflammatory mediators and lung permeability (series 2)

Inflammatory mediators for IFN-γ, IL-1β, IL-4, IL-6, IL-10, IL-13, IL-17A, TNF-α, KC and MIP-2 were analyzed in cell-free BAL and serum with a MSD U-plex® multiplex assay (Meso Scale Discovery®, Rockville, USA) while IL-22 was assessed in cell-free BAL and lung homogenate using a commercial ELISA kit (IL-22 Duoset DY582, Bio-techne Abingdon UK). Lung permeability was assessed via measurement of total protein concentration in cell-free BAL using the Bradford method (Bio-Rad, Temse, Belgium) and surfactant protein (SP)-D (Duoset ELISA R&D Systems Inc., DY6839-05) in the serum.

#### Flow cytometry analysis of mediastinal lymph nodes (series 2)

Single-cell suspensions were prepared from mediastinal lymph nodes by mechanical disruption. Cells were stained with conjugated antibodies directed against CD3, CD4, CD8α, CD44, CD62L and CD69 (eBioscience, Thermo Fisher Scientific). Dead cells were excluded from analysis following staining using Zombie Aqua Fixable Viability dye (BioLegend). Samples (> 100,000 cells) were acquired on a BD Canto II (BD Biosciences) flow cytometer, and data were analyzed with FlowJo software (Tree Star Inc., Ashland, OR).

#### Immunoglobulin production in serum and BAL (both mouse series and patient study)

In the mouse studies, mouse IgA, M and G in cell-free BAL and serum were measured via ELISA’s (Ready-Set-Go, Invitrogen, Merelbeke, Belgium). In the post-hoc analysis of the patient study, human IgG was measured in serum via ELISA (Ready-Set-Go, Invitrogen, Merelbeke, Belgium). All assays were performed according to manufacturer’s instructions. For the indirect ELISA against NTHi and DNA, validation of this home-made assay was first addressed by ensuring the absence of reaction against the blocking agent BSA and the serum and by using a positive control consisting in sample of NTHi infected mice for comparison. This permits to ensure that the reaction obtained with the samples of the study was surely due to binding with the NTHi antigen coated on the plates. Indirect ELISA against NTHi and DNA (calf thymus activated, Sigma-Aldrich), were performed by coating 50 μl/well of 10 μg/ml antigen in PBS. 96-well plates were incubated for 1 h at 37 °C. Plates were washed 3 times with PBS-Tween 0.05% and blocked with 150 μl PBS-BSA 1% for 1 h at 37 °C. Plates were washed 3 times and incubated with 1:4 dilution of BAL fluid or 1:32 dilution of serum samples. An arbitrary standard curve was made from pooled BAL fluid or serum samples with a dilution from 1:2 to 1:128. Samples were incubated for 2 h at 37 °C, washed 3 times and incubated for 1 h at room temperature with anti-mouse IgA, IgM and IgG biotinylated (Sigma-Aldrich) for the mice studies or anti-human IgG biotinylated (Sigma-aldrich) for human study. Plates were washed 3 times and incubated for 30 min with Streptavidin-HRP. TMB and stop solution was used for coloration and measured at 450 nm.

### Statistical analysis

Datasets were analyzed using GraphPad Prism 8.1.1 and are presented as median ± IQR. Population distribution, for each dataset, was examined with the Shapiro–Wilk normality test. For the animal studies: if the dataset was non-parametrically distributed, it was transformed with Log10 to a parametric dataset and further analyzed as a Two-way ANOVA with a Bonferroni *post-hoc* test for group comparison. In the post-hoc analysis of the patient study: baseline characteristics were analyzed using an unpaired T-test or Mann–Whitney test for parametric or non-parametric distributions. For the subgroup analysis dividing the participants into severe vitamin D deficient (< 10 ng/ml 25-OHD), vitamin D deficient (< 20 ng/ml) and vitamin D insufficient (< 30 ng/ml) patients, datasets with two variables were transformed, if non-normally distributed, with Log10 to a parametric dataset and analyzed with a Mixed-effects model for missing values and a Bonferroni *post-hoc* group comparison. Correlations were performed with a Spearman correlation between (1) ∆anti-NTHi antibodies and serum 25-OHD levels measured after one year of supplementation; (2) NTHi specific IgG (baseline, 12 months and gain) and the number of exacerbations and hospitalizations. This was performed with the total population and in the insufficient subgroup. A Poisson regression analysis was performed between the exacerbation/year and the ∆anti-NTHi antibodies. Differences were considered significant when p-value was less than 0.05.

## Results

### Mouse studies

#### Series 1

##### Serum measurements

25-OHD serum levels in VDD mice were < 2 ng/ml while in VDS mice 25-OHD levels were 20.8 ± 1.5 and 18.0 ± 3.3 ng/ml (mean ± SD) for the control and CS exposed mice, respectively.

##### Cell count in bronchoalveolar lavage

**Fig. 2:**
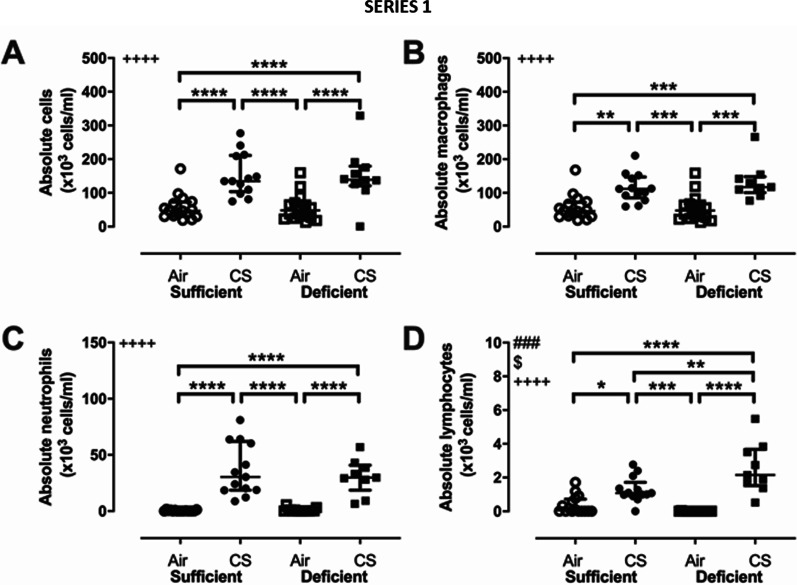
Acquired cellular inflammation measured in bronchoalveolar lavage after 14 weeks of CS exposure (series 1): **A** Absolute cells, **B** macrophages, **C** neutrophils and **D** lymphocytes in BAL fluid. ^###^p < 0.001 (two-way ANOVA, interaction); ^$^p < 0.05, (two-way ANOVA, sufficient vs deficient); ^++++^p < 0.0001 (two-way ANOVA, air vs CS); *p < 0.05, **p < 0.01, ***p < 0.001 ****p < 0.0001 (Bonferroni post-hoc test). Data are expressed as median ± IQR, with n = 8–15 mice/group

The impact of CS and VDD on the immune cell composition in BAL was first addressed in non-infected animals. Cigarette smoke exposure was associated with a significant increase in cellular inflammation in the lungs, as shown by the increase number of absolute cells, macrophages, neutrophils and lymphocytes in BAL fluid of the CS exposed mice compared to air exposed animals (Fig. 2A–D). In addition, the number of lymphocytes in mice exposed to CS was significantly higher in VDD compared to VDS mice (Fig. 2D). These findings show that being VDD accentuates cellular inflammation.

##### Immunoglobulin production in BAL and serum

**Fig. 3:**
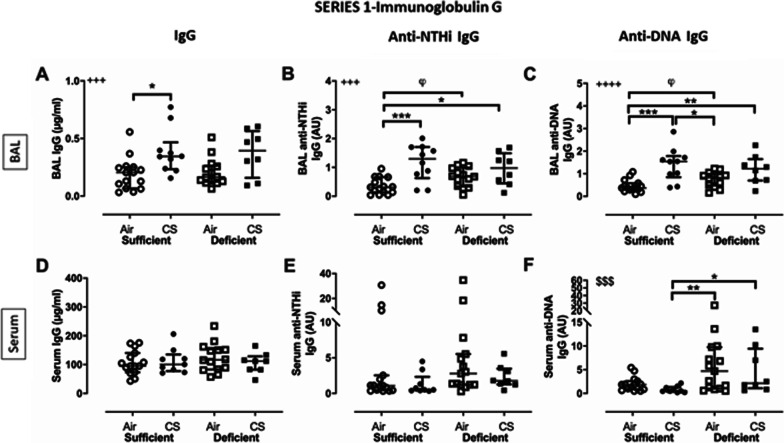
Production of total, anti-NTHi and anti-dsDNA immunoglobulin (Ig) G in BAL (**A**–**C**) and serum (**D**–**F**) in mice either vitamin D sufficient or deficient exposed to cigarette smoke or room air for 14 weeks (series 1). Total IgG (**A**, **D**) is expressed as µg/ml. Anti-NTHi and anti-dsDNA IgG are expressed in arbitrary units. ^$$$^p < 0.001 (two-way ANOVA, sufficient vs deficient); ^+++^p < 0.001, ^++++^p < 0.0001 (two-way ANOVA, air vs CS); *p < 0.05, **p < 0.01, ***p < 0.001 (Bonferroni post-hoc test) and φ: p < 0.05 (unpaired T-test, sufficient vs deficient). Data are expressed as median ± IQR, with n = 8–15 mice/group

To address the role of CS or VDD on adaptive immune response, total production of the different Ig’s but also specific production of Ig’s directed to NTHi and dsDNA were assessed in BAL and serum. Total IgA (Additional file 1: Fig. S1A) and IgG in BAL were significantly enhanced in CS compared to air exposed mice (Fig. 3A; 2-way ANOVA, p < 0.001) with no impact of vitamin D status while total IgM was undetectable. In serum, while total IgA and IgG were unchanged (Fig. 3D and Additional file 1: Fig. S1D), IgM showed a trend to be higher in VDD compared to VDS mice (Additional file 2: Fig. S2A), independently of smoking status. Production of anti-NTHi IgA (Additional file 1: Fig. S1B) and anti-NTHi IgG (Fig. 3B) in BAL was higher in CS exposed than air-exposed mice, and even in VDD air-exposed mice (Mann–Whitney, p < 0.05, Additional file 1: Figure S1B for anti-NTHi IgA and Fig. 3B for anti-NTHi IgG). In serum, production of anti-NTHi IgA (Additional file 1: Fig. S1E), anti-NTHi IgM (Additional file 2: Fig. S2B) and anti-NTHi IgG (Fig. 3E) was similar between the groups. Finally, natural IgA and IgG directed against DNA were significantly higher in BAL of CS exposed mice (Additional file 1: Fig. S1C and Fig. 3C) and increased levels of anti-dsDNA IgA, IgM and IgG (Additional file 1: Fig. S1F, Additional file 2: Fig. S2C and Fig. 3F) were found in serum of VDD mice. These findings indicate that anti-NTHi Ig’s are present within the lung prior to any NTHi infection and particularly under VDD and CS conditions.

#### Series 2

##### Serum measurements

25-OHD serum levels in VDD mice were < 2 ng/ml which is under the detection limit of LC–MS/MS measurement while 25-OHD serum levels in VDS mice averaged 25.9 ± 1.1 and 25.9 ± 3.5 ng/ml (Mean ± SD) in air and CS-exposed mice, respectively.

##### Pulmonary disease

**Fig. 4:**
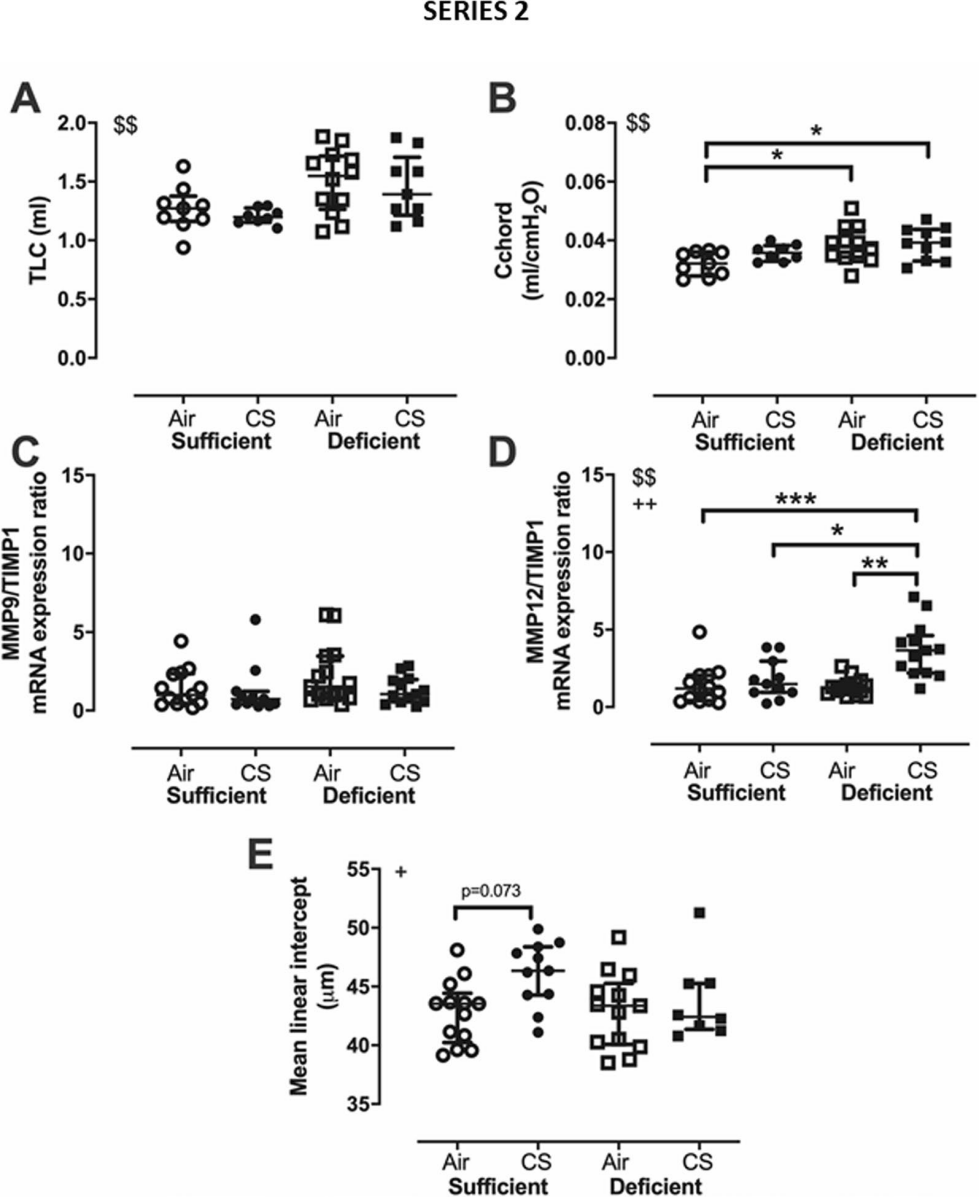
Lung function and remodeling in mice either vitamin D sufficient or deficient, repeatedly infected with NTHi and exposed either to CS or room air for 14 weeks (series 2). Lung function parameters **A** total lung capacity, TLC and **B** lung compliance, C chord. mRNA expression of **C** MMP9/TIMP1 and **D** MMP12/TIMP1 ratio in lung homogenate relative to RPL27. **E** Airspace enlargement measured via mean linear intercept. ^$$^p < 0.01 (two-way ANOVA, sufficient vs deficient); ^+^p < 0.05, ^++^p < 0.01, (two-way ANOVA, air vs CS); *p < 0.05, **p < 0.01, ***p < 0.001 (Bonferroni post-hoc test). Data are expressed as median ± IQR, with n = 9–15 mice/group

To address the impact of VDD on top of CS during repeated infections with NTHi on pulmonary disease, lung function, markers of lung proteolysis and lung morphology were assessed. For lung function, TLC increased significantly in VDD mice compared to VDS mice, independently of cigarette smoking (p = 0.003, 2-way ANOVA) (Fig. 4A). Cchord in VDD air- (+ 19%, p = 0.045) and CS-exposed (+ 22%, p = 0.032) mice was significantly enhanced in comparison with VDS air-exposed mice (Fig. 4B). For lung proteolysis, MMP9 mRNA expression remained similar between groups (Additional file 3: Fig. S3A). By contrast, MMP12 mRNA expression was significantly enhanced in the CS-exposed groups (p < 0.0001, 2-way ANOVA) and an interaction effect of VDD and CS exposure (p = 0.011, 2-way ANOVA) was shown with a 3.5-fold increase in VDD CS- compared to VDD air-exposed mice (p < 0.0001) (Additional file 3: Fig. S3B). Tissue MMP inhibitor 1 (TIMP1) mRNA expression was significantly reduced in VDD mice (p = 0.0073, 2-way ANOVA) (Additional file 3: Fig. S3C). No effect was observed on the MMP9/TIMP1 ratio (Fig. 4C), while MMP12/TIMP1 ratio was solely enhanced in the VDD CS-exposed mice (Fig. 4D). Finally, airspaces were significantly enlarged in the CS-exposed animals (2-way ANOVA, p = 0.03), with Lm being solely elevated in the VDS CS- (7%, p = 0.073) compared to VDS air-exposed animals (Fig. 4E). Taking together, these data show that VDD worsened lung function impairment and promoted lung proteolysis.

##### Bacterial clearance and subsequent inflammation

**Fig. 5:**
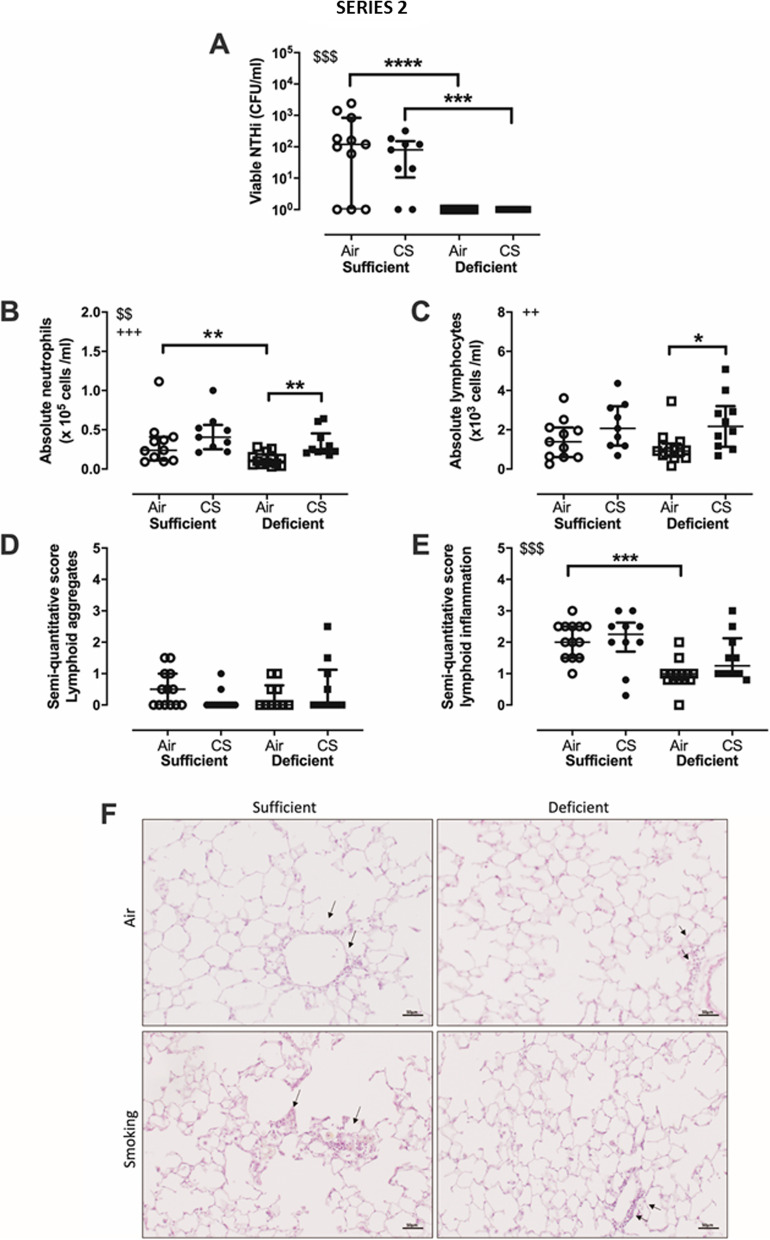
Bacterial clearance and acquired airway inflammation measured in broncho-alveolar lavage fluid (BAL) of mice either vitamin D sufficient or deficient, repeatedly infected with NTHi and exposed either to CS or room air for 14 weeks (series 2). **A** Colony forming units (CFU) of NTHi in BAL. Absolute number of **B** neutrophils and **C** lymphocytes in BAL. Semi-quantitative scoring of **D** lymphoid aggregates and **E** diffuse lymphoid inflammation in the lungs. **F** Representative H&E sagittal lung slides in each group showing an example of lymphoid inflammation indicated by arrows. ^$$^p < 0.01, ^$$$^p < 0.001 (two-way ANOVA, sufficient vs deficient); ^++^p < 0.01, ^+++^p < 0.001 (two-way ANOVA, air vs CS); *p < 0.05, **p < 0.01, ***p < 0.001, ****p < 0.0001 (Bonferroni post-hoc test). Data are expressed as median ± IQR, with n = 8–15 mice/group

To determine whether VDD would worsen CS-induced bacterial eradication and alterations in immune cell composition during repeated infections with NTHi, bacterial clearance was assessed in BAL and inflammation was measured in BAL and lung. The NTHi infection was completely eradicated in the BAL of VDD mice compared to VDS mice (p = 0.0001, 2-way ANOVA), whereas 72–77% of the VDS mice remained infected 72 h post-infection, independently of CS (Fig. 5A). As for the absolute cell number in BAL (data not shown), CS significantly increased the number of neutrophils (p = 0.0003, 2-way ANOVA) and lymphocytes (p = 0.0038, 2-way ANOVA) while both were reduced in VDD air-exposed mice compared to the other groups (Fig. 5B-C). Lymphoid aggregation in the lungs was moderate with no differences between groups (Fig. 5D, F). On the other hand, lung inflammation was mainly driven by a diffuse lymphoid inflammation, which was lower in VDD mice compared to VDS and significantly lower in VDD air- (p = 0.0004) compared to VDS air-exposed mice (Fig. 5E, F). Collectively, these findings indicated that VDD enhanced NTHi eradication and reduced lung inflammation.

##### Pro-inflammatory mediators

As both CS and VDD are known to affect epithelial barrier integrity, total protein concentration in BAL and serum levels of SP-D were measured upon bacterial challenge. In addition, their potential role in exacerbating repeated NTHi-mediated cytokine production in lung and serum was determined. Total protein concentration in BAL and SP-D levels in serum were similar between the different groups (Additional file 4: Fig. S4A, Fig. S4B). Pro-inflammatory cytokines e.g. TNF-α, IFN-γ, IL-17a, IL-10 and IL-6 in the lungs were significantly lower in VDD mice compared to VDS (Additional file 10: Table S3) while IL-4 and IL-13 fell below detection limit (data not shown). No differences were found in pro-inflammatory cytokines TNF-α and IL-10 in serum whereas IL-4, IL-13, IL-6 and IL-1β fell below detection limit. Compared to VDS, serum levels of IFN-γ and IL-17A were significantly lower in VDD mice (respectively p = 0.0002; p = 0.0097; 2-way ANOVA). IL-17A increased significantly with CS (p = 0.0006; 2-way ANOVA) being significantly higher in VDD CS- compared to VDD air-exposed mice (p = 0.0046). KC and MIP-2 decreased significantly in BAL of VDD mice and even more in VDD air-exposed mice while they remained unchanged in serum (Additional file 10: Table S3). These data indicated that chronic inflammation was located in the lung and resolved after the NTHi infection in VDD mice. As IL-22 plays an essential role in the clearance of NTHi, IL-22 was measured in BAL and lung homogenate. While IL-22 was below detection limit in BAL, its concentration in lung was similar between the different groups (Additional file 10: Table S3).

##### Lymph node activation

To determine how CS and VDD would affect immune cell activation in response to repeated infections with NTHi, immune cell profile was examined in lymph node. VDD mice showed a higher percentage of CD3+ cells within the total lymphocyte gate in mediastinal lymph nodes compared to their respective VDS group (Additional file 5: Fig. S5A), with no difference in CD4+/CD8+ ratio (Additional file 5: Fig. S5B). VDS and VDD mice showed similar percentages of total CD4+ effector memory cells (defined as CD44^hi^CD62L^hi^) (Additional file 5: Fig. S5C), however, in VDD mice these cells showed a less activated phenotype measured by a smaller CD69 + activation with CS (p = 0.001, 2-way ANOVA) and in VDD mice (p = 0.006, 2-way ANOVA) (Additional file 5: Fig. S5D). The frequency of CD8+ effector memory (CD44^hi^CD62L^hi^) cells was significantly reduced in VDD mice compared to VDS mice (p = 0.0004, 2-way ANOVA) (Additional file 5: Fig. S5E). Interestingly, CS-exposed mice had a higher percentage of CD8+ effector memory cells compared to their respective air-exposed group (Additional file 5: Fig. S5E). CD8+ effector memory cells showed a higher CD69+ activation in VDD mice than VDS mice (p = 0.02, 2-way ANOVA) (Additional file 5: Fig. S5F). Taking together, these findings show that a resolution of effector memory CD4+ and CD8+ cells is occurring in VDD, but CD8+ effector memory cells are still highly active.

### Immunoglobulin production in BAL and serum

**Fig. 6:**
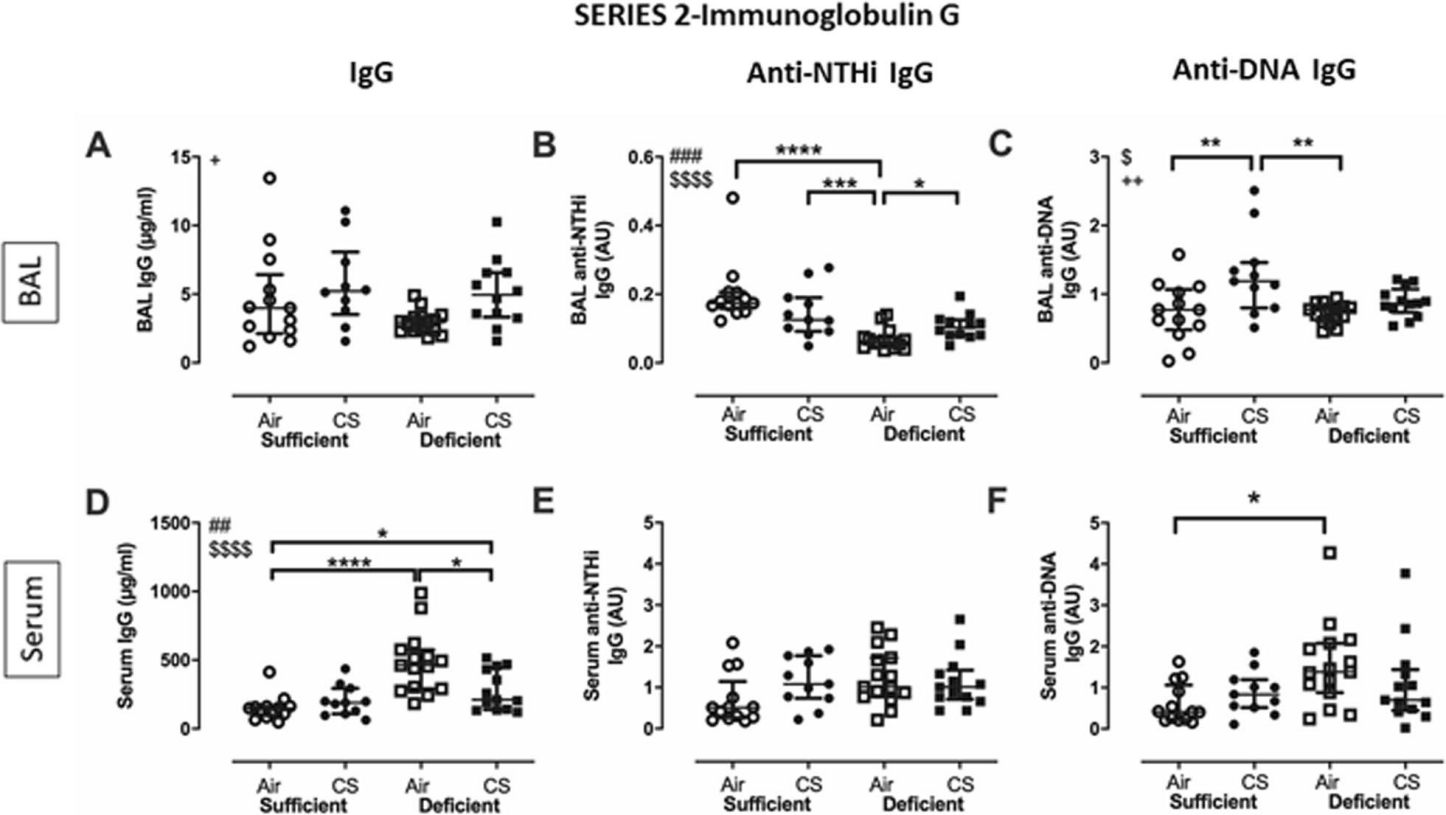
Production of total, anti-NTHi and anti-dsDNA immunoglobulin (Ig) G in BAL (**A**–**C**) and serum (**D**–**F**) of mice either vitamin D sufficient or deficient, repeatedly infected with NTHi and exposed either to CS or room air for 14 weeks (series 2). Total IgG (**A** and **D**) is expressed as µg/ml. Anti-NTHi IgG (**B** and **E**) and anti-dsDNA IgG (**C** and **F**) are expressed in arbitrary units.^##^p < 0.01, ^###^p < 0.001 (two-way ANOVA, interaction); ^$^p < 0.05, ^$$$$^p < 0.0001 (two-way ANOVA, sufficient vs deficient); *p < 0.05, **p < 0.01, ***p < 0.001 ****p < 0.0001 (Bonferroni post-hoc test). Data are expressed as median ± IQR, with n = 9–15 mice/group

The role of CS or VDD during bacterial challenge on adaptive immune response has been addressed by measuring in BAL and serum total Ig production as well as specific production of Ig’s directed against NTHi and dsDNA. Production of total IgA, IgM and IgG in BAL was decreased in VDD air-exposed mice compared to VDS-air and VDD-CS (Additional file 6: Fig. S6A, Additional file 7: Fig. S7A, Fig. 6A). While serum levels of IgA remained unchanged (Additional file 6: Fig. S6D), IgM and IgG serum levels were significantly increased in VDD air- compared to VDS air- (p = 0.030 and p < 0.0001, respectively) and VDD CS-exposed mice (p = 0.056 and p = 0.032, respectively) (Additional file 7: Fig. S7D, Fig. 6D). Anti-NTHi IgA and anti-NTHi IgG decreased in BAL of VDD-air exposed mice compared to VDS air- (p = 0.0019 and p < 0.0001, respectively) and VDD CS-exposed mice (p = 0.018 and p = 0.030, respectively) (Additional file 6: Fig. S6B, Fig. 6B), with no changes observed in BAL anti-NTHi IgM levels (Additional file 7: Fig. S7B). In serum, only anti-NTHi IgA slightly increased in VDD-CS compared to VDS-air (p = 0.022) and VDD-air (p = 0.0063) exposed mice (Additional file 6: Fig. S6E) while anti-NTHi IgM and anti-NTHi IgG remained stable (Additional file 7: Fig. S7E, Fig. 6E). Lastly, CS exposure resulted in enhanced levels of natural IgA and IgG direct against DNA in BAL (Additional file 6: Fig. S6C, Fig. 6C) and in serum, VDD air-exposed mice showed increased levels of anti-dsDNA IgM and anti-dsDNA IgG (Additional file 7: Fig. S7F, Fig. 6F) while VDS CS-exposed mice displayed higher levels of serum anti-dsDNA IgA (Additional file 6: Fig. S6F). Collectively, these data show an enhanced resolution of Ig’s in BAL of VDD and an increase in serum with an affinity for dsDNA instead of NTHi.

## Patient study

### Baseline characteristics

**Fig. 7:**
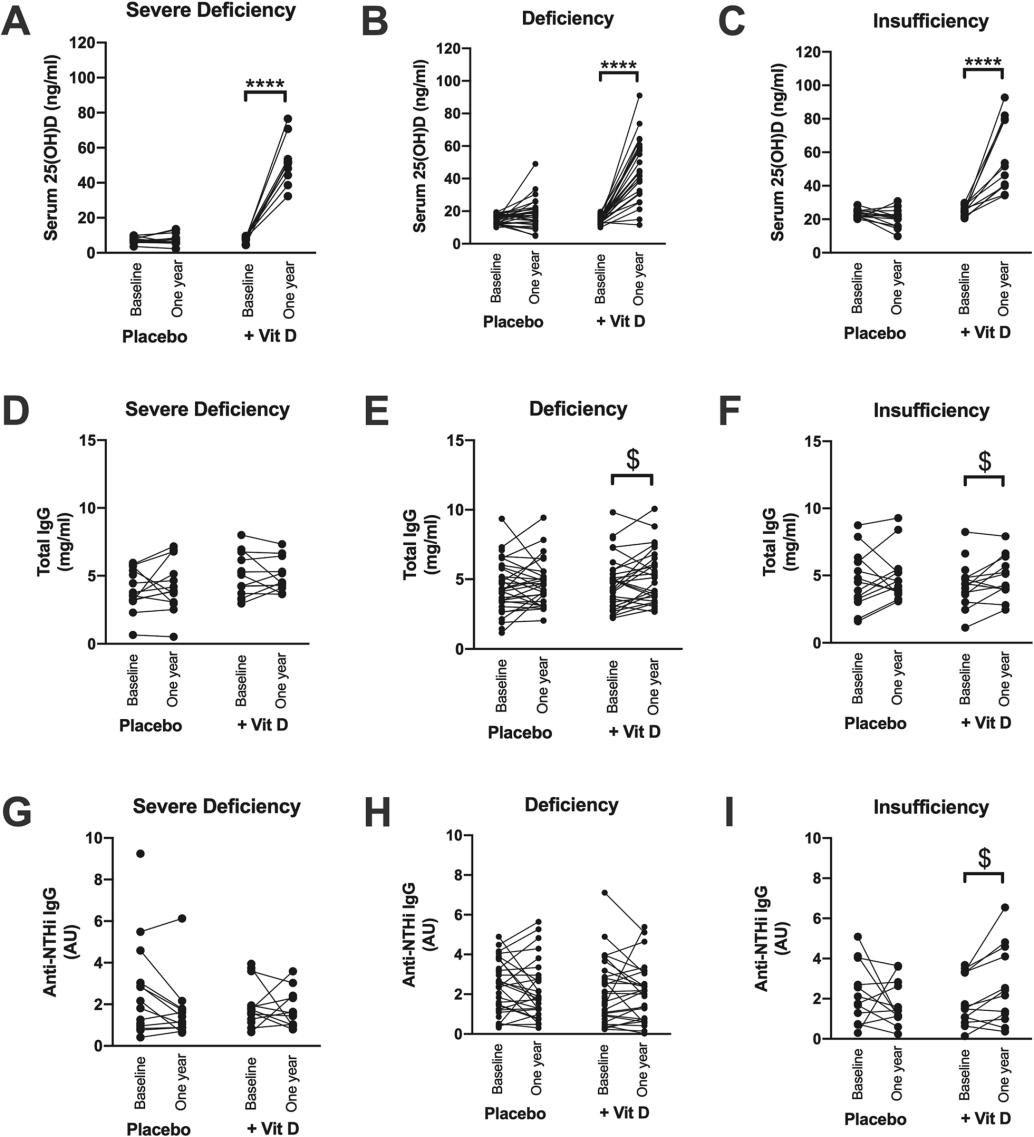
Serum 25-OHD levels (**A**–**C**), total IgG (**D**–**F**) and anti-NTHi IgG (**G**–**I**) in COPD patients treated with placebo or vitamin D for one year. Patients are subdivided according to their baseline levels of serum 25-OHD in severe deficiency (< 10 ng/ml serum 25OHD), deficiency (< 20 ng/ml) and insufficiency (< 30 ng/ml). Each point represents an individual patient. Serum levels of 25-OHD are expressed in ng/ml. Total IgG are expressed as mg/ml. Anti-NTHi IgG are expressed in arbitrary units (AU). ****p < 0.0001 (Mixed effect analysis with Bonferroni post-hoc test); ^$^p < 0.05 (Paired T-test or Wilcoxon matched pairs)

Enrolled COPD patients were divided in a placebo (n = 67) and vitamin D3 (n = 70) treated groups. There were no differences in gender, smoking status, GOLD stage, lung function and 25-OHD serum levels between both groups. When the patients were subdivided according to their 25-OHD serum levels, no differences in 25-OHD serum levels were found between the placebo and vitamin D treated group regarding the vitamin D severely deficient (mean ± SD: 7.5 ± 1.8 vs 8.0 ± 1.6 ng/ml, respectively) (Fig. 7A), deficient (14.7 ± 2.8 vs 14.5 ± 2.4 ng/ml, respectively) (Fig. 7B), insufficient (23.4 ± 2.4 vs 24.6 ± 3.0 ng/ml, respectively) (Fig. 7C) and sufficient (40.3 ± 6.8 vs 38.5 ± 8.7 ng/ml, respectively) COPD patients.

#### Serum levels of 25-OHD evolution

Serum levels of 25-OHD in patients enrolled in the placebo group remained stable after one year of treatment, while it significantly increased towards sufficient 25-OHD levels (> 30 ng/ml) in the vitamin D3 treated COPD patients (Fig. 7A–C). The median rise in 25-OHD levels show non-significant higher gain in the more severe vitamin D deficient group, meaning that the intervention is successful in reaching sufficient 25-OHD levels in each group independently of the levels of vitamin D deficiency at baseline (Additional file 8: Fig. S8).

### Production of total IgG in serum

To determine whether vitamin D3 treatment could potentially boost baseline IgG serum levels, total IgG was measured in serum of the placebo or vitamin D treated COPD patients. At baseline, total IgG in serum was similar between the different groups (Fig. 7D–F). After one year, while total IgG in serum remained unchanged in the placebo-treated group, it significantly increased after vitamin D3 treatment in the patients with vitamin D deficiency and insufficiency (Fig. 7D-E).

#### Production of anti-NTHi and anti-dsDNA IgG in serum

To determine whether COPD patients produce anti-NTHi and anti-dsDNA Ig’s and whether these levels are affected by serum levels of vitamin D, anti-NTHi and anti-dsDNA IgG were measured in serum. At baseline, production of anti-NTHi IgG (Fig. 7G–I) and anti-dsDNA IgG (Additional file 9: Fig. S9A–C) in serum was similar between the different subgroups and did not significantly change over time in the placebo group. At the end of the study, serum anti-NTHi IgG (Fig. 7G and H) and serum anti-dsDNA IgG (Additional file 9: Fig. S9A and B) of severely vitamin D deficient and vitamin D deficient COPD patients were not affected by vitamin D supplementation. This was also the case for serum anti-dsDNA in vitamin D insufficient patients (Additional file 9: Fig. S9C) in whom, however, serum anti-NTHi IgG significantly increased after vitamin D supplementation (p = 0.012, Fig. 7I). No correlation was found between serum anti-NTHi IgG at baseline, 12 months or its gain and the number of exacerbations and the number of hospitalizations for the entire population but also for the vitamin D deficient and severely deficient COPD patients. Only a moderate inverse relationship was observed between anti-NTHi IgG at 12 months and the number of exacerbations for the vitamin D insufficient COPD group (r = − 0.47, p < 0.01). There was no correlation between endpoint 25-OHD serum levels and the gain in anti-NTHi IgG between baseline and 12 months for the entire population (r = 0.19; p = 0.05). This correlation was only observed in a subgroup of patients with insufficient 25-OHD serum levels at baseline (r = 0.46, p = 0.022). Finally, in the whole population, a 10.4% decrease in anti-NTHi IgG would increase the probability for one extra exacerbation/year (p = 0.004). Taking together, these data indicate that vitamin D3 supplementation can increase antibody production but only in vitamin D insufficient COPD patients at baseline. However, anti-NTHi IgG in serum are needed in reducing the exacerbation frequency.

## Discussion

In contrast to our hypothesis, vitamin D deficiency did neither promote inflammation upon CS exposure nor prolong the NTHi infections during repeated infections in mice. In fact, vitamin D deficiency enhanced the eradication of NTHi, independently of CS exposure, and promoted lung proteolysis by shifting the protease/anti-protease balance towards more proteolysis. Interestingly, Ig’s were increased in BAL of CS and VDD mice included antibodies to NTHi and DNA even before infection with NTHi. This spontaneous polyclonal antibody production could explain the boost in eradication in VDD mice when exposed to repeated NTHi infections. However, VDD show increased levels of anti-DNA Ig serum in both non-and NTHi infected mice. In contrast, in COPD patients one-year treatment with vitamin D3 was not associated with an increase in anti-NTHi or anti-DNA IgG production in serum but it did result in enhanced anti-NTHi IgG in serum of COPD patients who were vitamin D insufficient at baseline.

The lower airways of the lungs have always been perceived to be sterile, but advancements in molecular techniques (e.g. 16s rRNA sequencing) have shown the presence of a microbiome in healthy individuals consisting out of Proteobacteria, Firmicutes, Bacteroidetes and Actinobacteria [34]. This microbiome is most likely introduced via micro-aspirations that transport bacteria from the upper to the lower airways during breathing [35]. As COPD progresses, environmental and structural changes of the airways will cause a subtle change of the microbiome and immunity. The harsher environment (e.g. oxidative stress, pH, mucus, humoral mediators, etc.) and defective immunity give room for natural selection and change the normal lung microbiome to a less diverse with more Proteobacteria and Actinobacteria [34, 36]. In COPD, opportunistic bacteria become more virulent and colonize the lower airways and, once abundant, they could cause repeated exacerbations whether or not from the same strain (36, 37). Forty to 60% of the exacerbations are driven by bacterial infections [38] with *NTHi, S. pneumonia, M. catarrhalis* and *P. aeruginosa* being the most commonly isolated bacteria from sputum samples during exacerbations [39]. Animal studies have shown that CS-exposed animals are more susceptible to environmentally acquired bacterial infections (e.g. *Pneumocytis murina*) [40]. Similarly, CS-exposed mice subjected to a single intra-nasal instillation of NTHi and *S. pneumonia* demonstrated colonization of the upper respiratory tract and even translocation of *S. pneumonia* into the lungs [41]. Taken together, these data suggest that cigarette smoking can affect the lower airway colonization and infections in COPD patients, but other factors such as vitamin D deficiency could play a role as well.

Several studies have associated vitamin D deficiency as a risk factor for disease progression in COPD patients [8–10, 26, 27]. Still it remains difficult to draw firm conclusions from these observational and randomized control studies concerning the exact role of vitamin D deficiency in inducing lung function impairment and especially in promoting exacerbations. Therefore, animal models can offer the advantage of exploring potential causality while highlighting the specific contribution of vitamin D deficiency to COPD exacerbation. In a previous study, we found that VDD mice were more susceptible to lung remodeling after a single infection with NTHi by expressing more MMP12 [32]. This led to a shift in the protease/anti-protease balance towards more proteolysis during the infection period [32]. We postulated that this could potentially damage the lungs especially when presented with repeated infections in such a way that it would exacerbate disease progression when combined with CS-exposure. Interestingly, in the current study, hyperinflation of the lungs with increased lung compliance were solely observed after repeated NTHi infections in VDD mice, regardless of CS-exposure. These data support the idea that vitamin D deficiency by itself makes the lung more vulnerable to repeated infections promoting worsening of lung function. On the other hand, the interaction effect found between vitamin D deficiency and CS exposure on MMP12 and imbalance of MMP12/TIMP1 ratio towards proteolysis in smoking and VDD animals support the postulate that vitamin D deficiency would promote lung damage during the course of repeated infection especially when combined with CS.

Surprisingly, in the current study, NTHi was eradicated faster in VDD mice than in VDS mice. This is in agreement with our previous data after a single NTHi infection [32]. In the current study, this faster eradication of NTHi was, however, not mediated through enhanced levels of IL-22 in VDS mice. To our knowledge, the current study is the first to report the effects of repeated airway infections of viable NTHi in vitamin D deficiency and in combination with CS. Only a few studies with CS exposure have investigated the effects of repeated infections in mice. Gou et al showed a twofold higher bacterial burden in mice exposed to CS for 20 weeks which were monthly instilled with *S. pneumoniae* [42]. On the other hand, combined CS-exposure with already heat-killed NTHi infections demonstrated only a mild lung inflammatory response, but enlarged airspaces compared to non-infected CS exposed animals [43]. Therefore, it is still surprising to find this faster eradication of NTHi in VDD mice and no increased bacterial burden in CS-exposed mice.

Data in VDR^−/−^ mice shows alterations in tight and adherens junctions in the epithelial and endothelial layer of the lungs indicating that pulmonary barrier integrity is weakened and this may lead to increased lung permeability and risk for oedema [17]. In the current study, no differences in lung permeability were observed three days after the last infection with NTHi, since neither protein concentration in BAL nor serum levels of SP-D differ between the groups. This is in agreement with our single infection study with NTHi where no oedema and no changes in BAL protein levels were found between VDD and VDS mice [32]. Discrepancies with the data in VDR^−/−^ mice might be related to differences in infectious doses where pneumonia or an acute respiratory distress syndrome is induced rather than a lower respiratory tract infection. Moreover, our NTHi-infected mice did not show any systemic inflammation, indicating that the infection was only localized in the lungs.

CS exposure has the potential to reduce the generation of CD4+ and CD8+ effector memory T-lymphocytes [44, 45], but this was not shown in response to repeated NTHi infections in our study. Instead, similar amounts of CD4+ effector memory cells were generated in all groups, but with less activation (CD69 +) in VDD mice which could be attributed to the complete eradication of NTHi at sacrifice. Similarly, CS did not reduce the generation of CD8+ effector memory cells but show less CD8+ effector memory cells in VDD mice and could be in the contraction phase. VDR^−/−^ mice infected with a lymphocytic choriomeningitis virus, showed a less broadened repertoire of antigen-specific effector memory CD8+ T cells and demonstrated an enhanced contraction phase compared with wild type mice due to the decrease in the prosurvival expression of Bcl-2 [46]. Strangely, we found unlike the CD4+ effector memory cells that CD8+ effector memory cells in VDD showed a slightly higher frequency of the CD69+ activation marker. Knowing that the NTHi infection in our model is not invasive, making thus the CD8+ lymphocyte immune branch obsolete, we postulate that this higher activation could participate in the development of self-antigen recognition T-cells and contribute to the autoimmune branch in COPD. It is therefore possible that these cells will act against own lung tissue and account for the ongoing lung destruction after smoking cessation.

Although some studies have provided evidences for an impaired adaptive immunity with CS (reviewed in [47, 48], we surprisingly found in BAL and not in serum an increase in IgA and IgG after CS. In addition, we show that these Ig have an affinity towards NTHi but interestingly in the absence of any previous NTHi infection. It is therefore possible that Ig provoked by CS could demonstrate a cross reactivity for NTHi or CS could carry antigens from NTHi knowing that bioactive LPS is present in CS [49]. Intriguingly, an increase in anti-NTHi IgA and anti-NTHi IgG was also found in VDD mice neither exposed to CS or NTHi infection. This higher production of immunoglobulins within the lung, at baseline, might provide an explanation for the faster eradication of NTHi in VDD mice and such a trend with CS. We already reported faster NTHi eradication after only one infection with NTHi [32], which supports the concept of the presence of baseline anti-NTHi Ig’s in the lungs. However, in the repeatedly NTHi-infected VDS mice, there were no increases in anti-NTHi IgG and anti-NTHi IgA with cigarette smoking, indicating that repeated infections on top of cigarette smoking disturbed the production of anti-NTHi Ig induced by cigarette smoking alone. It is, however, unknown how repeated infections is masking the effect of cigarette smoking on anti-NTHi Ig production. Unfortunately, we were not able to confirm a higher anti-NTHi IgG production in vitamin D deficient COPD patients because the original study was not designed for that purpose with all the participants being vitamin D insufficient at baseline. However, our data indicated beneficial increases in total IgG in serum one year after vitamin D supplementation in vitamin D deficient and insufficient COPD patients. For the latter, higher anti-NTHi IgG could potentially protect against an exacerbation, as only a 10.4% decrease in these anti-NTHi IgG could provoke one more extra exacerbation/year in the studied population. Strangely, these effects were not found in severely vitamin D deficient and vitamin D deficient COPD patients suggesting that epigenetic alterations are probably involved due to prolonged vitamin D deficiency exposure. We speculate that a longer time of stable 25-OHD serum levels is needed to resolve these attenuated effects on the immunity.

In the study with repeated NTHi infections, the complete clearance of NTHi with VDD was reflected by the reduced levels of total Ig’s and anti-NTHi Ig’s in BAL while serum immunoglobulin titers, which usually remain longer at steady-state, are still higher within VDD. Strangely, these Ig’s showed only little affinity for NTHi, but higher affinity towards DNA. Indeed, VDD was associated with elevated production of anti-dsDNA Ig’s in BAL or in serum in the absence of any infection but also after repeated infections with NTHi. Anti-dsDNA Ig’s are natural anti-nuclear autoantibodies (ANA) that are generated during infections and in some autoimmune diseases (e.g. systemic lupus erythematosus (SLE), rheumatoid arthritis (RA) and Hashimoto thyroiditis (HT)) [50–52]. It is still unknown how these Ig’s are produced, as it could be due to molecular mimicry of bacterial/viral surface proteins, or lingering bacterial DNA seen as an antigen, which is strange because DNA is an adjuvant for the innate immunity [53]. Furthermore, studies in SLE, RA and HT have reported a negative correlation with vitamin D serum levels in these autoimmune diseases [50–52]. In COPD patients these anti-dsDNA IgG are also found and could have destructive consequences for the lung [54]. Anti-dsDNA Ig’s have a diverse recognition of DNA e.g. single-stranded DNA, DNA/RNA hybrids etc. but also show a cross-reactivity with annexin II, α-actinin, laminin, collagen III, collagen IV, entactin, complement receptor type 1 (C1q) N-methyl-d-aspartate receptor, ribosomal P proteins, heparin sulfate etc. [55]. This potential cross-reactivity of anti-dsDNA IgG with some proteins of the extracellular matrix could further stimulate lung destruction or even a self-perpetuated autoimmune reaction in the lungs of COPD patients. Interestingly, these anti-DNA IgG’s were also present in the serum of the COPD patients although no correlation was found. Our data also underlined that these baseline anti-DNA IgG’s were not increasing synergistically with the increase in total IgG and anti-NTHi after vitamin D3 supplementation. Altogether, vitamin D3 treatment could potentially boost the immune system in reducing bacterial exacerbations in the long run.

This study explores for the first time the effect of repeated infections of viable NTHi in a previously established CS exposure mouse model with severe vitamin D deficiency [19]. The NTHi strain used in this study was isolated from a sputum sample of a COPD patient going through an exacerbation. Previously, we showed the virulent properties of this NTHi strain in inducing an inflammatory response in mice lungs [32]. Nevertheless, several limitations should be acknowledged. Firstly, instillation of the mice with NTHi took place at a specific location and were quarantined for one week, due to health safety environmental legislation of our institution. During this period, mice could not be exposed to CS and could have influenced our findings, as CS exposure inhibits phagocytosis in alveolar macrophages and neutrophils. For example, neutrophils can restore there phagocytic ability already 40 h post-infection [32]. Secondly, the VDD mouse model we used, is not fully translatable to humans with regards to the immunological processes. Mice do not react similarly and some vitamin D responsive elements in genes are missing e.g. cathelicidin, which is an antimicrobial peptide directed against gram negative bacteria such as NTHi. Finally, exacerbations in COPD are caused by a diversity of bacterial strains and viruses, but mostly by a virulent strain that colonizes the lower respiratory tract.

## Conclusion

While vitamin D deficiency worsened lung function impairment during repeated infections with NTHi and promoted autoantibodies production pre- and post-infection, it also expedited the clearance of NTHi from the lungs and the resolution of local lung inflammation. In COPD patients, the increase in total IgG and anti-NTHi IgG in vitamin D deficient and for the latter also in vitamin D insufficient patients, after supplementation with vitamin D3, suggests a protective role of vitamin D against exacerbations in this patient population, without worsening autoimmunity complications. Future research is necessary to explore the dual effect of vitamin D for either being protective or self-destructive and the potential beneficial effects against exacerbations in a larger group of COPD patients.

## Supplementary Information

**Additional file 1: Figure S1.** Production of total, anti-NTHi and anti-dsDNA immunoglobulin (Ig) A in BAL (A-C) and serum (D-F) in mice either vitamin D sufficient or deficient exposed to cigarette smoke or room air for 14 weeks (series 1). Total IgA (A, D) is expressed as µg/ml, anti-NTHi and anti-dsDNA IgA are expressed in arbitrary units. $: p < 0.05, $$$$: p < 0.0001 (two-way ANOVA, sufficient vs deficient); ++++: p<0.0001 (two-way ANOVA, air vs CS); *: p < 0.05, **: p < 0.01, ***: p < 0.001 ****: p < 0.0001 (Bonferroni post-hoc test) and φ: p < 0.05 (unpaired T-test, sufficient vs deficient). Data are expressed as median ± IQR, with n = 8-15 mice/group.

**Additional file 2: Figure S2.** Production of total (A), anti-NTHi (B) and anti-dsDNA (C) IgM in serum of mice either vitamin D sufficient or deficient exposed to cigarette smoke or room air for 14 weeks (series 1). Total IgM (A) is expressed as µg/ml. Anti-NTHi (B) and anti-dsDNA IgM (C) are expressed in arbitrary units. $: p < 0.05 (two-way ANOVA, sufficient vs deficient); +: p < 0.05 (two-way ANOVA, air vs CS); *: p < 0.05, **: p < 0.01 (Bonferroni post-hoc test). Data are expressed as median ± IQR, with n = 8-15 mice/group

**Additional file 3: Figure S3.** mRNA expression of lung MMP9 (A), MMP12 (B) and TIMP1(C) relative to RPL27 in mice either vitamin D sufficient or deficient exposed to cigarette smoke or room air for 14 weeks (series 2). $ p < 0.05, $$: p < 0.01 (two-way ANOVA, sufficient vs deficient); ++++: p < 0.0001 (two-way ANOVA, air vs CS); * p < 0.05, **: p < 0.01, ****: p < 0.0001 (Bonferroni post-hoc test). Data are expressed as median ± IQR, with n = 9-15 mice/group.

**Additional file 4: Figure S4.** Protein concentration in BAL (A) fluid and SP-D serum levels (B) of mice either vitamin D sufficient or deficient exposed to cigarette smoke or room air for 14 weeks (series 2). Protein concentration is expressed as µg/ml. $ p < 0.05, (two-way ANOVA, sufficient vs deficient). Data are expressed as median ± IQR, with n = 9-15 mice/group.

**Additional file 5: Figure S5.** Lymphocyte differentiation in mediastinal lymph nodes of mice either vitamin D sufficient or deficient exposed to cigarette smoke or room air for 14 weeks (series 2). (A) Lymphocytes were characterized with flow cytometry (B) CD3+ lymphocytes, (C) ratio of CD4+/CD8+ lymphocytes, (D) CD4+ T-effector memory (TEM) (CD44hiCD62Llow) with its (E) activation marker (CD69+) and (F) CD8+ TEM (CD44hiCD62Llow) with its (G) activation marker (CD69+). Data are expressed as percentage of parent. $: p < 0.05, $$: p < 0.01; $$$: p < 0.001 (two-way ANOVA, sufficient vs deficient); +: p < 0.05, +++: p < 0.001 (two-way ANOVA, air vs CS); *: p < 0.05, ***: p < 0.001, (Bonferroni post-hoc test). Data are expressed as median ± IQR, with n = 8-9 mice/group.

**Additional file 6: Figure S6.** Production of total, anti-NTHi and anti-dsDNA IgA in BAL (A-C) and serum (D-F) of mice either vitamin D sufficient or deficient exposed to cigarette smoke or room air for 14 weeks (series 2). Total IgA (A, D) is expressed as µg/ml. Anti-NTHi and anti-dsDNA IgA are expressed in arbitrary units. #: p < 0.05, ##: p < 0.01 (two-way ANOVA, interaction); $: p < 0.05, $$: p < 0.01 (two-way ANOVA, sufficient vs deficient); +: p < 0.05, +++: p < 0.001, (two-way ANOVA, air vs CS); *: p < 0.05, **: p < 0.01 (Bonferroni post-hoc test). Data are expressed as median ± IQR, with n = 9-15 mice/group.

**Additional file 7: Figure S7.** Production of total, anti-NTHi and anti-dsDNA IgM in BAL (A-C) serum (D-F) of mice either vitamin D sufficient or deficient exposed to cigarette smoke or room air for 14 weeks (series 2). Total IgM (A, D) is expressed as µg/ml. Anti-NTHi (B, E) and anti-dsDNA IgM (C, F) are expressed in arbitrary units. ##: p < 0.01 (two-way ANOVA, interaction);  $: p < 0.05 (two-way ANOVA, sufficient vs deficient); *: p < 0.05, **: p < 0.01 (Bonferroni post-hoc test). Data are expressed as median ± IQR, with n = 9-15 mice/group.

**Additional file 8: Figure S8.** Rise in 25-OHD serum levels of COPD patients supplemented with vitamin D3 at the end of the study. Baseline 25-OHD serum levels were severely deficient in 12 patients (<10ng/ml), deficient in 28 (<20ng/ml) and insufficient in 12 (<30ng/ml) patients. 25-OHD serum levels are expressed as ng/ml. Data is expressed as median±IQR.

**Additional file 9: Figure S9.** Post-hoc subgroup analysis of COPD-patients in the placebo and vitamin D supplementation group for serum production of anti-dsDNA IgG in (A) severely deficient (<10ng/ml), (B) deficient (<20ng/ml) and (C) insufficient (<30ng/ml) levels of 25-OHD. Data at baseline are compared with data after one year supplementation with placebo or with vitamin D3. Each point represents an individual patient. Data is expressed in arbitrary units (AU).

**Additional file 10: Table S1.** Primer sequences (Series 2). **Table S2.** Baseline characteristics of the COPD patients in the placebo and vitamin D supplemented group. **Table S3.** Pro-inflammatory mediators in lung homogenate and serum of mice either vitamin D sufficient or deficient, repeatedly infected with NTHi and exposed either to CS or room air for 14 weeks (series 2)

## Data Availability

All data generated or analyzed during this study are included in this published article and its supplementary information files. Datasets used or analyzed during the current study are available from the corresponding author on reasonable request.

## Abbreviations

COPD: Chronic obstructive pulmonary disease
NTHi: Non-typeable *Heamophilus influenzae*
VDS: Vitamin D sufficient
VDD: Vitamin D deficient
CS: Cigarette smoke
Ig: Immunoglobulin
TLC: Total lung capacity
Cchord: Chest wall compliance
25-OHD: 25-Hydroxyvitamin D
VDR: Vitamin D receptor
LPS: Lipopolysaccharide
MMP-12: Matrix metalloproteinase-12
TIMP1: Tissue MMP inhibitor 1
CFU: Colony forming units
i.p.: Intraperitoneally
BAL: Broncho-alveolar lavage
RCT: Random controlled trial
FEV1: Forced expiratory volume in 1 s
FVC: Forced vital capacity
Lm: Mean linear intercept
SP-D: Surfactant protein-D
