# Supplementary figures and images for: Effects of repeated infections with non-typeable Haemophilus influenzae on lung in vitamin D deficient and smoking mice
Source: Respir Res. 2022 Mar 2;23:40. doi: 10.1186/s12931-022-01962-6 (PMC8889723; doi:10.1186/s12931-022-01962-6)

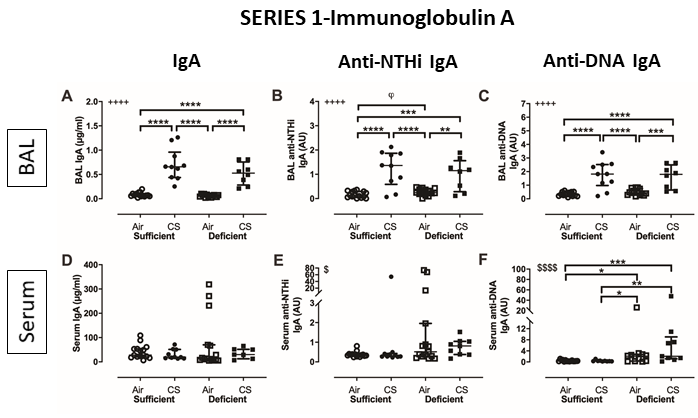

Supplement: Supplementary file 1 — Additional file 1: Figure S1. Production of total, anti-NTHi and anti-dsDNA immunoglobulin (Ig) A in BAL (A-C) and serum (D-F) in mice either vitamin D sufficient or deficient exposed to cigarette smoke or room air for 14 weeks (series 1). Total IgA (A, D) is expressed as µg/ml, anti-NTHi and anti-dsDNA IgA are expressed in arbitrary units. $: p < 0.05, $$$$: p < 0.0001 (two-way ANOVA, sufficient vs deficient); ++++: p<0.0001 (two-way ANOVA, air vs CS); *: p < 0.05, **: p < 0.01, ***: p < 0.001 ****: p < 0.0001 (Bonferroni post-hoc test) and φ: p < 0.05 (unpaired T-test, sufficient vs deficient). Data are expressed as median ± IQR, with n = 8-15 mice/group. [file 12931_2022_1962_MOESM1_ESM.tif]

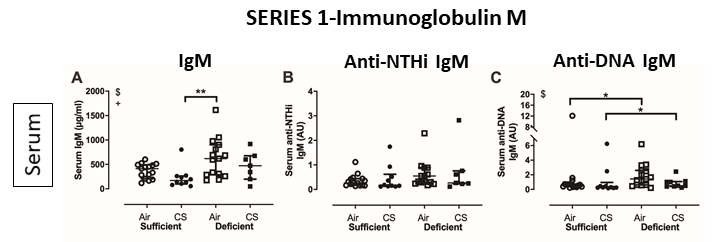

Supplement: Supplementary file 2 — Additional file 2: Figure S2. Production of total (A), anti-NTHi (B) and anti-dsDNA (C) IgM in serum of mice either vitamin D sufficient or deficient exposed to cigarette smoke or room air for 14 weeks (series 1). Total IgM (A) is expressed as µg/ml. Anti-NTHi (B) and anti-dsDNA IgM (C) are expressed in arbitrary units. $: p < 0.05 (two-way ANOVA, sufficient vs deficient); +: p < 0.05 (two-way ANOVA, air vs CS); *: p < 0.05, **: p < 0.01 (Bonferroni post-hoc test). Data are expressed as median ± IQR, with n = 8-15 mice/group [file 12931_2022_1962_MOESM2_ESM.tif]

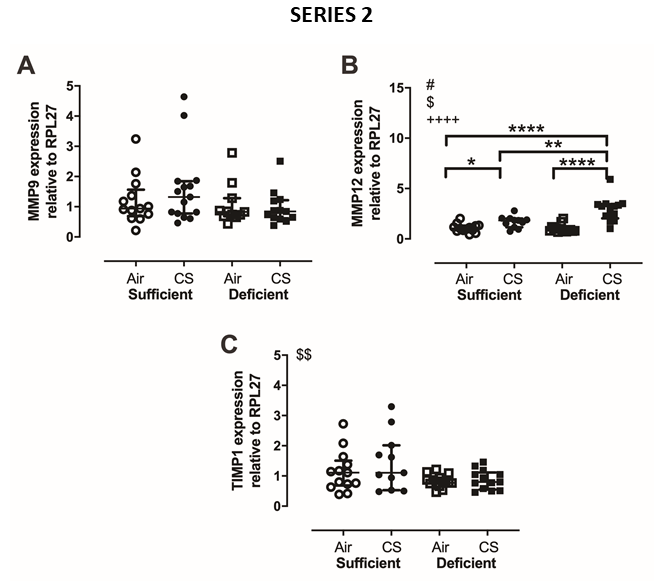

Supplement: Supplementary file 3 — Additional file 3: Figure S3. mRNA expression of lung MMP9 (A), MMP12 (B) and TIMP1(C) relative to RPL27 in mice either vitamin D sufficient or deficient exposed to cigarette smoke or room air for 14 weeks (series 2). $ p < 0.05, $$: p < 0.01 (two-way ANOVA, sufficient vs deficient); ++++: p < 0.0001 (two-way ANOVA, air vs CS); * p < 0.05, **: p < 0.01, ****: p < 0.0001 (Bonferroni post-hoc test). Data are expressed as median ± IQR, with n = 9-15 mice/group. [file 12931_2022_1962_MOESM3_ESM.tif]

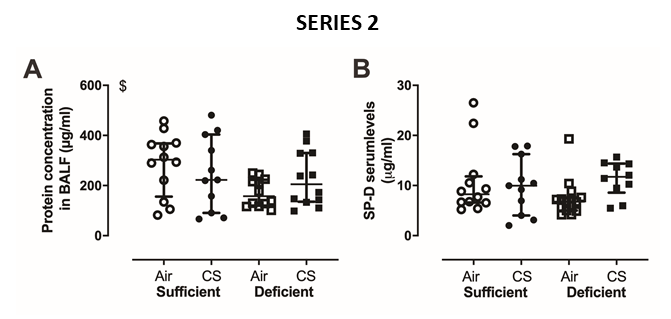

Supplement: Supplementary file 4 — Additional file 4: Figure S4. Protein concentration in BAL (A) fluid and SP-D serum levels (B) of mice either vitamin D sufficient or deficient exposed to cigarette smoke or room air for 14 weeks (series 2). Protein concentration is expressed as µg/ml. $ p < 0.05, (two-way ANOVA, sufficient vs deficient). Data are expressed as median ± IQR, with n = 9-15 mice/group. [file 12931_2022_1962_MOESM4_ESM.tif]

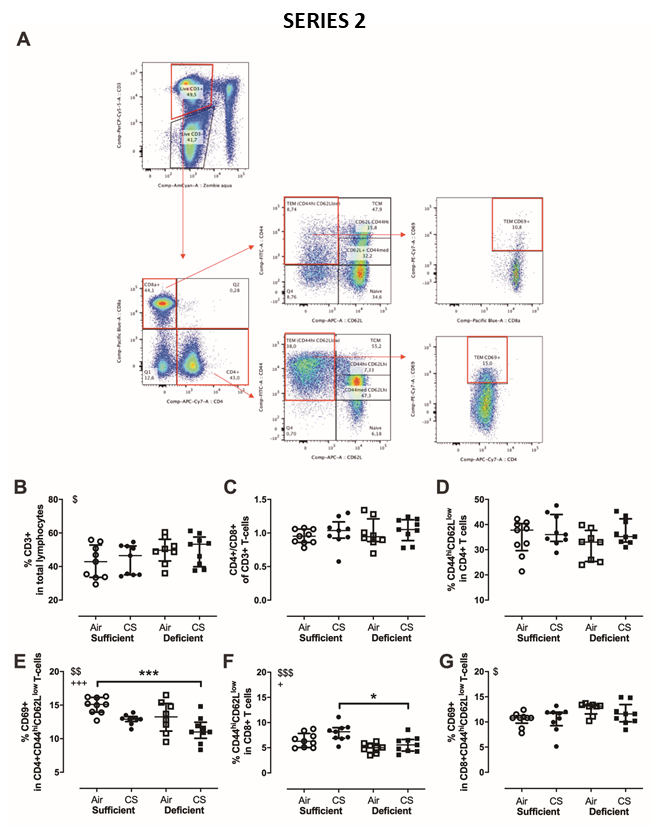

Supplement: Supplementary file 5 — Additional file 5: Figure S5. Lymphocyte differentiation in mediastinal lymph nodes of mice either vitamin D sufficient or deficient exposed to cigarette smoke or room air for 14 weeks (series 2). (A) Lymphocytes were characterized with flow cytometry (B) CD3+ lymphocytes, (C) ratio of CD4+/CD8+ lymphocytes, (D) CD4+ T-effector memory (TEM) (CD44hiCD62Llow) with its (E) activation marker (CD69+) and (F) CD8+ TEM (CD44hiCD62Llow) with its (G) activation marker (CD69+). Data are expressed as percentage of parent. $: p < 0.05, $$: p < 0.01; $$$: p < 0.001 (two-way ANOVA, sufficient vs deficient); +: p < 0.05, +++: p < 0.001 (two-way ANOVA, air vs CS); *: p < 0.05, ***: p < 0.001, (Bonferroni post-hoc test). Data are expressed as median ± IQR, with n = 8-9 mice/group. [file 12931_2022_1962_MOESM5_ESM.tif]

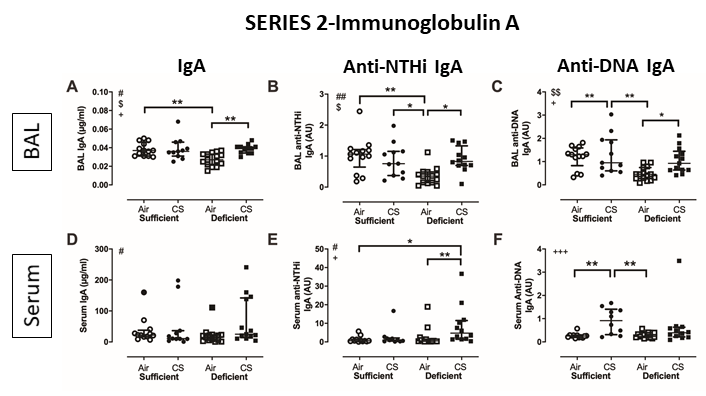

Supplement: Supplementary file 6 — Additional file 6: Figure S6. Production of total, anti-NTHi and anti-dsDNA IgA in BAL (A-C) and serum (D-F) of mice either vitamin D sufficient or deficient exposed to cigarette smoke or room air for 14 weeks (series 2). Total IgA (A, D) is expressed as µg/ml. Anti-NTHi and anti-dsDNA IgA are expressed in arbitrary units. #: p < 0.05, ##: p < 0.01 (two-way ANOVA, interaction); $: p < 0.05, $$: p < 0.01 (two-way ANOVA, sufficient vs deficient); +: p < 0.05, +++: p < 0.001, (two-way ANOVA, air vs CS); *: p < 0.05, **: p < 0.01 (Bonferroni post-hoc test). Data are expressed as median ± IQR, with n = 9-15 mice/group. [file 12931_2022_1962_MOESM6_ESM.tif]

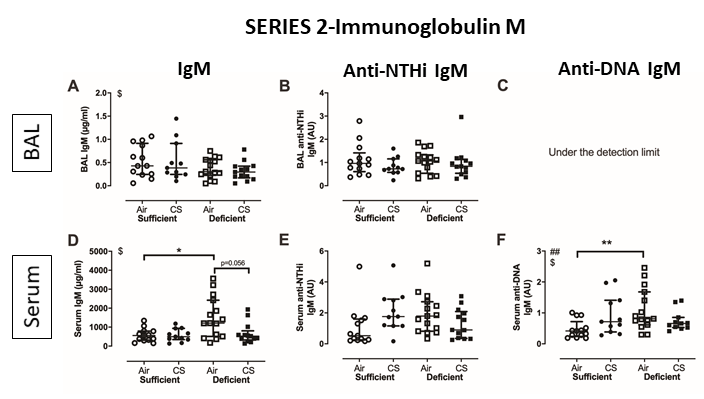

Supplement: Supplementary file 7 — Additional file 7: Figure S7. Production of total, anti-NTHi and anti-dsDNA IgM in BAL (A-C) serum (D-F) of mice either vitamin D sufficient or deficient exposed to cigarette smoke or room air for 14 weeks (series 2). Total IgM (A, D) is expressed as µg/ml. Anti-NTHi (B, E) and anti-dsDNA IgM (C, F) are expressed in arbitrary units. ##: p < 0.01 (two-way ANOVA, interaction); $: p < 0.05 (two-way ANOVA, sufficient vs deficient); *: p < 0.05, **: p < 0.01 (Bonferroni post-hoc test). Data are expressed as median ± IQR, with n = 9-15 mice/group. [file 12931_2022_1962_MOESM7_ESM.tif]

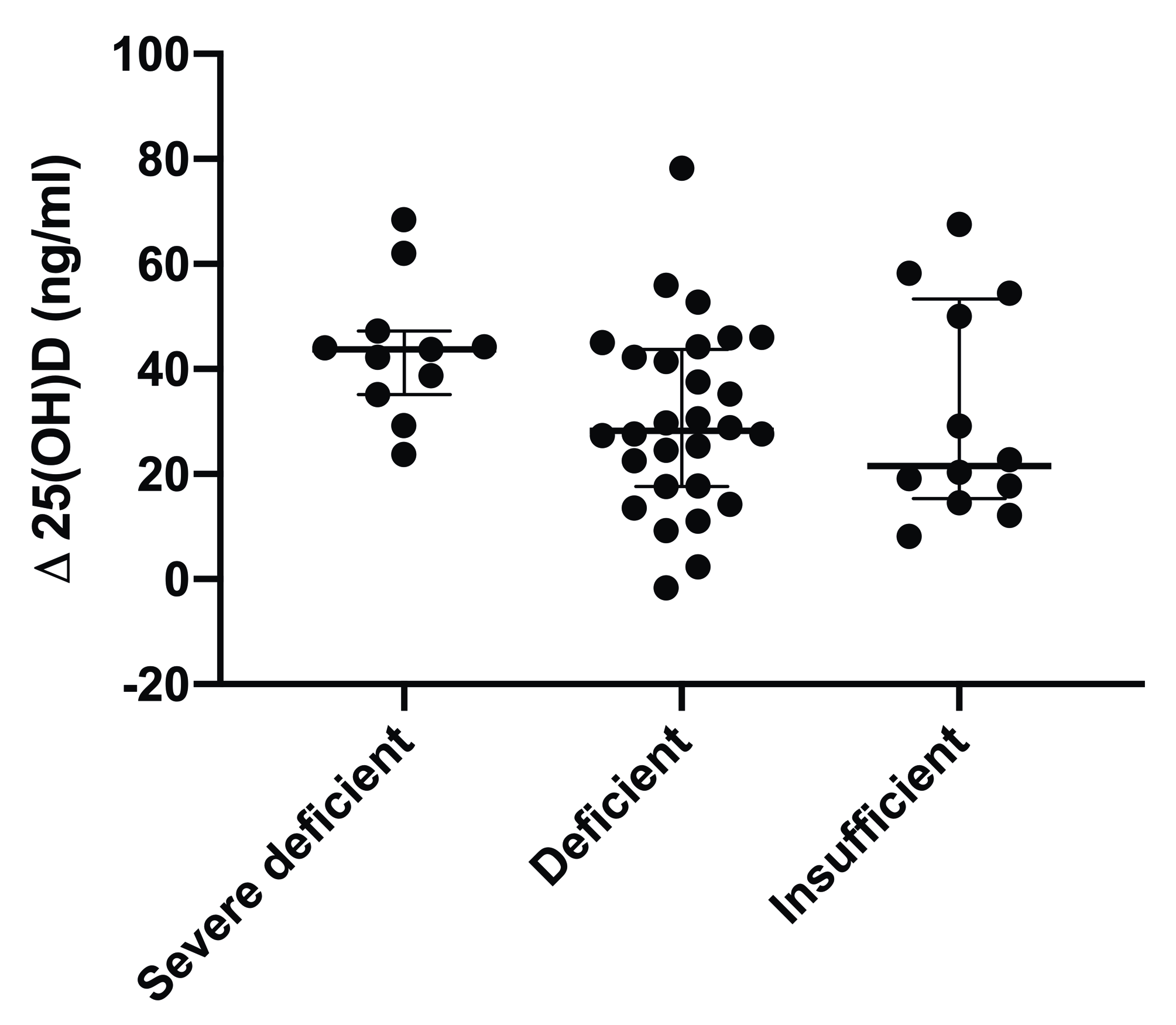

Supplement: Supplementary file 8 — Additional file 8: Figure S8. Rise in 25-OHD serum levels of COPD patients supplemented with vitamin D3 at the end of the study. Baseline 25-OHD serum levels were severely deficient in 12 patients (<10ng/ml), deficient in 28 (<20ng/ml) and insufficient in 12 (<30ng/ml) patients. 25-OHD serum levels are expressed as ng/ml. Data is expressed as median±IQR. [file 12931_2022_1962_MOESM8_ESM.tiff]

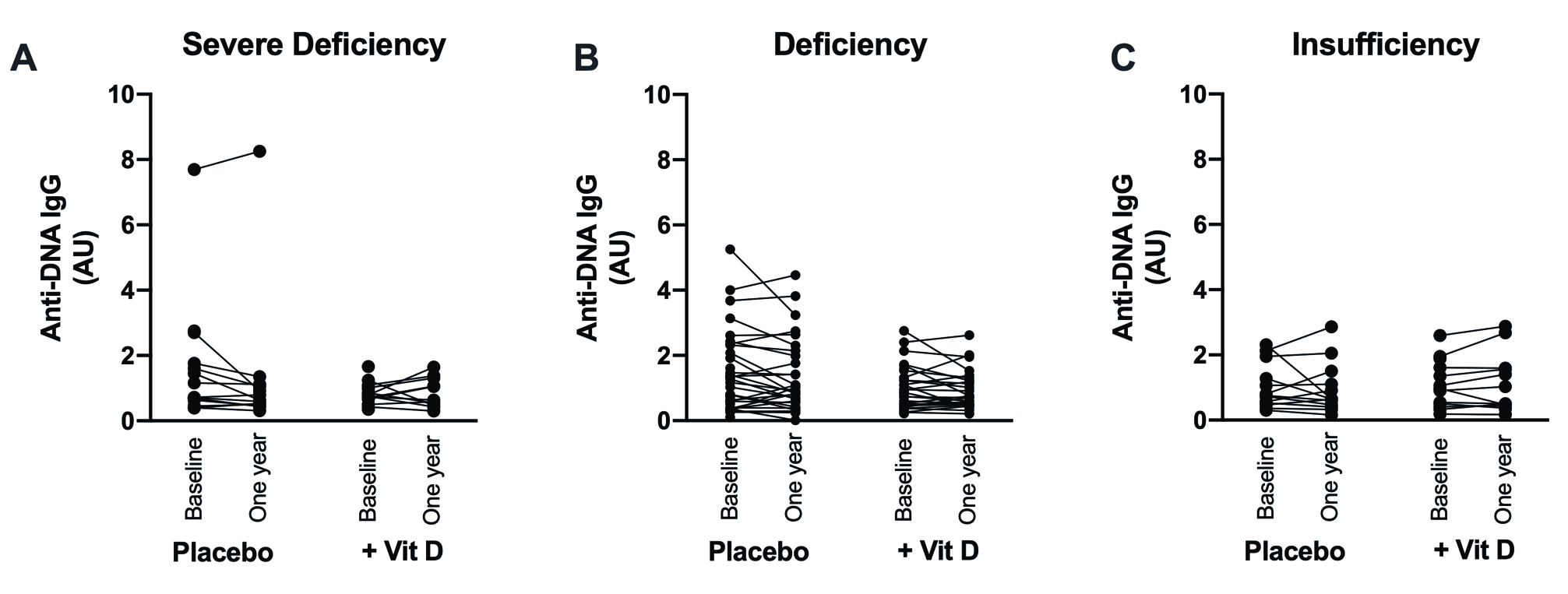

Supplement: Supplementary file 9 — Additional file 9: Figure S9. Post-hoc subgroup analysis of COPD-patients in the placebo and vitamin D supplementation group for serum production of anti-dsDNA IgG in (A) severely deficient (<10ng/ml), (B) deficient (<20ng/ml) and (C) insufficient (<30ng/ml) levels of 25-OHD. Data at baseline are compared with data after one year supplementation with placebo or with vitamin D3. Each point represents an individual patient. Data is expressed in arbitrary units (AU). [file 12931_2022_1962_MOESM9_ESM.tiff]
