# Supplementary material for: Effects of repeated infections with non-typeable Haemophilus influenzae on lung in vitamin D deficient and smoking mice
Source: Respir Res. 2022 Mar 2;23:40. doi: 10.1186/s12931-022-01962-6 (PMC8889723; doi:10.1186/s12931-022-01962-6)
Supplement: Supplementary file 10 — Additional file 10: Table S1. Primer sequences (Series 2). Table S2. Baseline characteristics of the COPD patients in the placebo and vitamin D supplemented group. Table S3. Pro-inflammatory mediators in lung homogenate and serum of mice either vitamin D sufficient or deficient, repeatedly infected with NTHi and exposed either to CS or room air for 14 weeks (series 2) [file 12931_2022_1962_MOESM10_ESM.docx]

## Table S1:

**Table S1:** Primer sequences (Series 2)

| Target | Forward primer | Reverse primer | Accession ID |
| --- | --- | --- | --- |
| RPL27 | 5’- GTCGAGATGGGCAAGTTCAT-3’ | 5’-TTCTTCACGATGACGGCTTT-3’ | NM_011289.3 |
| MMP12 | 5’-TTTTGATGGCAAAGGTGGTA-3’ | 5’-GCCTCATCAAAATGTGCATC-3’ | [NM_001320077.1 NM_001320076.1 NM_008605.3](https://www.ncbi.nlm.nih.gov/nucleotide/991820332?report=genbank&log$=nucltop&blast_rank=2&RID=SS462SGT01R) |
| MMP9 | 5’-TTCCCCAAAGACCTGAAAAC-3’ | 5’-TGCTTCTCTCCCATCATCTG-3’ | NM_013599.4 |
| TIMP1 | 5’-GTGGGAAATGCCGCAGAT-3’ | 5’-GGGCATATCCACAGAGGCTTT-3’ | [NM_001044384.1](https://www.ncbi.nlm.nih.gov/nucleotide/NM_001044384.1?report=genbank&log$=nucltop&blast_rank=3&RID=5UMMFX40014)  [NM_011593.2](https://www.ncbi.nlm.nih.gov/nucleotide/NM_011593.2?report=genbank&log$=nucltop&blast_rank=2&RID=5UMMFX40014)  [NM_001294280.2](https://www.ncbi.nlm.nih.gov/nucleotide/NM_001294280.2?report=genbank&log$=nucltop&blast_rank=1&RID=5UMMFX40014) |

Ribosomal protein L27 (RPL27), matrix metalloproteinases 9 (MMP9) and 12 (MMP12), tissue inhibitor of matrix metalloproteinase (TIMP1). All primers were certified with a primer efficiency in the range of 90-105%.

## Table S2:

**Table S2:** Baseline characteristics of the COPD patients in the placebo and vitamin D supplemented group

| Characteristics | Placebo group  n= 67 | Vitamin D group  n= 70 | P-value |
| --- | --- | --- | --- |
| Male n (%) | 59 (88.0) | 58 (82.8) |  |
| Female n (%) | 8 (12.0) | 12 (17.2) |  |
| Current smokers n (%) | 15 (22.4) | 11 (15.7) |  |
| GOLD I n (%) | 0 (0) | 1 (1.4) |  |
| GOLD II n (%) | 18 (26.9) | 21 (29.6) |  |
| GOLD III n (%) | 35 (53.5) | 32 (45.1) |  |
| GOLD IV n (%) | 14 (19.7) | 17 (23.9) |  |
| FEV1 L (% predicted) | 1.22±0.44 (43±14) | 1.25±0.45 (45±16) | >0.05 |
| FVC L (% predicted) | 2.95±0.88 (80±19) | 2.82±0.8 (79±21) | >0.05 |
| FEV1/FVC % | 42.09±11.28 | 44.41±12.05 | >0.05 |
| total population 25(OHD) ng/mL (mean±SD) | 16.05±7.71 | 16.8±8.29 | >0.05 |
| Severly deficient ng/ml (n) | 7.5±1.8 (15) | 8.0±1.6 (14) | >0.05 |
| Deficient ng/ml (N) | 14.7±2.8 (36) | 14.5±2.4 (38) | >0.05 |
| insufficient ng/ml (N) | 23.4±2.4 (13) | 24.6±3.0 (14) | >0.05 |
| sufficient ng/ml (N) | 40.3±6.8 (3) | 38.5±8.7 (4) | >0.05 |

### Table S3

**Table S3: Pro-inflammatory mediators in lung homogenate and serum of mice either vitamin D sufficient or deficient, repeatedly infected with NTHi and exposed either to CS or room air for 14 weeks (series 2)**

|  | **LUNG** | | | | **SERUM** | | | |
| --- | --- | --- | --- | --- | --- | --- | --- | --- |
|  | **Sufficient** | | **Deficient** | | **Sufficient** | | **Deficient** | |
|  | **AIR (pg/μg)** | **CS (pg/μg)** | **AIR (pg/μg)** | **CS (pg/μg)** | **AIR (pg/ml)** | **CS (pg/ml)** | **AIR (pg/ml)** | **CS (pg/ml)** |
| **IL-1b** | 356.8 (213.7-845.9) | 529.6 (162.7-716.2) | 107.7 (80.8-131.1)^bbb^ | 196.5 (131.1-231.3)^c^ | / | / | / | / |
| **TNF-⍺** | 74.5 (43.2-147.5) | 90.7 (47.5-113.5) | 27.4 (23.2-31.5)^bbb^ | 52.8 (27.4-67.0)^c^ | 10.0 (9.2-12.1) | 10.2 (8.8-11.5) | 9.3 (8.0-11.8) | 10.9 (9.4-12.3) |
| **IL-6** | 86.1 (38.8-179.2) | 66.5 (37.6-246.6) | 22.3 (11.2-27.5)^b^ | 42.3 (19.8-46.6) | / | / | / | / |
| **IFN-𝜸** | 5.5 (1.5-17.6) | 3.7 (1.5-24.3) | 1.1 (0.7-1.8)^b^ | 2.2 (0.7-2.9)^c^ | 1.3 (0.4-1.7) | 2.0 (0.7-3.2) | 0.3 (0.1-0.4)^bb^ | 0.5 (0.3-1.5) |
| **IL-17a** | 109.6 (93.1-157.9) | 112.1 (65.5-182.7) | 26.5 (22.3-44.2)^bbb^ | 71.2 (47.0-149.2) | 1.8 (1.2-2.0) | 2.2 (1.6-3.1) | 0.6 (0.5-0.8)^b^ | 1.7 (1.3-2.9)^aa^ |
| **IL-10** | 6.9 (2.7-12.0) | 8.8 (3.0-12.5) | 2.0 (0.9-2.9)^b^ | 4.5 (1.8-5.7) | 10.4 (8.6-18.5) | 8.9 (8.2-11.1) | 11.4 (9.2-16.7) | 9.3 (6.9-14.3) |
| **IL-22** | 1119 (813-1386) | 1131 (1059-1258) | 1189 (898-1239) | 1302 (901-1530) | NA | NA | NA | NA |
| **KC** | 388.6 (223.9-760.9) | 469.7 (204.6-800.6) | 171.5 (143.6-199.1)^bb^ | 360.2 (189.8-430.5) | 63.2 (39.5-94.9) | 79.9 (54.2-82.6) | 58.4 (42.5-79.2) | 71.3 (59.5-103.7) |
| **MIP-2** | 214.0 (95.9-529.9) | 218.1 (86.1-380.8) | 65.2 (46.9-71.7)^bb^ | 118.8 (77.7-157.4) | 22.4 (14.5-26.7) | 18.3 (16.7-21.5) | 18.7 (10.9-26.4) | 22.8 (17.7-27.6) |

Data are expressed as median (Q1-Q3)

^aa^: p<0.01, Deficient (Air vs CS)

^b^: p<0.05, ^bb^: p<0.01, ^bbb^: p<0.001 (Sufficient air vs Deficient air)

^c^: p<0.05 (Sufficient air vs Deficient CS)

/: below detection limit

NA not assessed

N=8-12/group
